# Synthesis of the pentasaccharide repeating unit of the *O*-antigen of *E. coli* O117:K98:H4

**DOI:** 10.3762/bjoc.10.287

**Published:** 2014-11-20

**Authors:** Pintu Kumar Mandal

**Affiliations:** 1 Medicinal and Process Chemistry Division, CSIR-Central Drug Research Institute, BS-10/1, Sector 10, Jankipuram extension, Sitapur Road, Lucknow, 226 031, India

**Keywords:** *Escherichia coli*, glycosylation, lipopolysaccharide, *O*-antigen, pentasaccharide

## Abstract

The pentasaccharide repeating unit of the *O*-antigen of *E. coli* O117:K98:H4 strain has been synthesized using a combination of sequential glycosylations and [3 + 2] block synthetic strategy from the suitably protected monosaccharide intermediates. Thioglycosides and glycosyl trichloroacetimidate derivatives have been used as glycosyl donors in the glycosylations.

## Introduction

**Figure 1:**
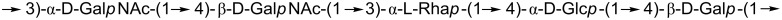
Structure of the pentasaccharide repeating unit of the *O*-specific polysaccharide of *E. coli* O117:K98:H4.

*Escherichia coli* becomes an important human pathogen in recent years owing to the emergence of new pathogenic strains [1]. Several diseases, such as meningitis and sepsis [2], diarrhoeal outbreaks [3] and urinary tract infections [4] are associated with pathogenic *Escherichia coli* (*E. coli*) strains. *E. coli* strains have been found to produce the Shiga toxin (Stx), heat-labile (LT) or heat-stable (ST) enterotoxins, cytotoxic necrotizing factors (CNF1 and CNF2) and hemolysins (α-Hly and E-Hly) [5–6] and are responsible for hemorrhagic colitis and haemolytic-uremic syndroms in humans [7]. The different strains of *E.coli* as well as bacteria belonging to different genera, e.g., *Shigella*, *Salmonella*, and *Klebsiella* show serological cross-reactions within the species [8]. The *E. coli* O117 strain emerged as a significant cause for septicaemia, bovine diarrhoea in new born children and human [9]. Together with other *E. coli* strains *E. coli* 0117 strains are responsible for pyelonephritis which is sexually transmitted by a woman that spread up to 60 to 80% of community acquired urinary tract or travelled through the bloodstream to the kidneys [10–11]. The *O*-specific polysaccharide of *E. coli* O117:K98:H4 is a linear pentasaccharide repeating unit consisting of D-galactosamine, D-glucose, D-galactose, and L-rhamnose (Figure 1) [12].

**Figure 2:**
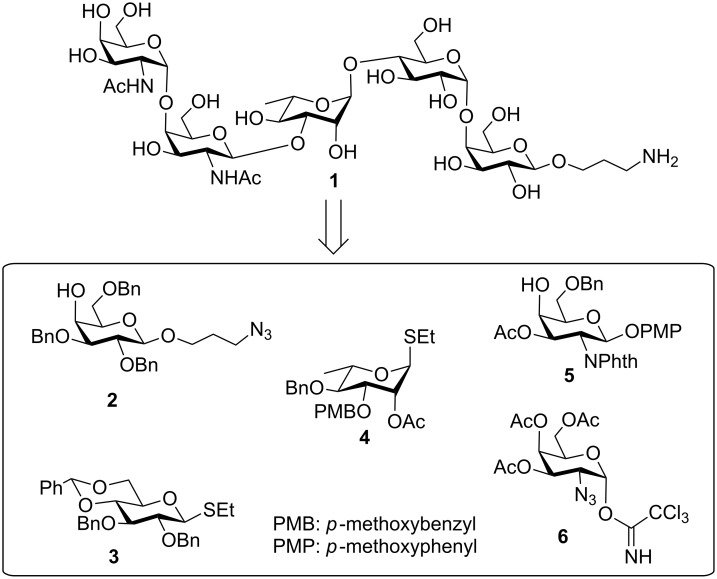
Structure of the synthesized pentasaccharide (**1**) and its precursor intermediates.

Vaccination is the recent thrust in the drug discovery program to prevent bacterial infections. Several bacterial *O*-antigens have been chosen for the development of glycoconjugate vaccine candidates against infectious diseases [13–16]. As a consequence, a significant quantity of oligosaccharides is required to evaluate their immunological properties for detailed understanding of the role of *O*-antigens in the pathogenicity of the *E. coli* strains. Development of chemical synthetic strategies would be useful to get large quantities of the oligosaccharides. As a part of the ongoing studies on the synthesis of bacterial cell wall oligosaccharides [17–19], a straightforward synthesis of the pentasaccharide repeating unit of the *O*-specific polysaccharide of *E. coli* O117:K98:H4 as its 3-aminopropyl glycoside is presented herein (Figure 2). The 3-aminopropyl group would be suitable for attachment of the pentasaccharide to any surface or carrier proteins.

## Results and Discussion

The target pentasaccharide **1** has been synthesized as its 3-aminopropyl glycoside using a combination of sequential and [3 + 2] block glycosylation strategy. A trisaccharide acceptor **11** and a disaccharide trichloroacetimidate donor **14** were synthesized from the appropriately protected monosaccharide intermediates **2** [20], **3** [21], **4** [22], **5** and **6** [23] (Figure 2) derived from the commercially available aldoses. Trisaccharide acceptor **11** was then glycosylated with disaccharide trichloroacetimidate donor **14** to form pentasaccharide derivative **15**, which was finally deprotected to give target pentasaccharide **1** (see below Scheme 2). Some of the notable features of this synthetic strategy are (a) application of iodonium ion mediated general glycosylation conditions; (b) nitrosyl tetrafluoroborate (NOBF_4_) mediated activation of glycosyl trichloroacetimidate donor; (c) the attachment of an aminopropyl linker at the anomeric center; (d) glycosylation and removal of the *p*-methoxybenzyl (PMB) group in one-pot.

**Scheme 1:**
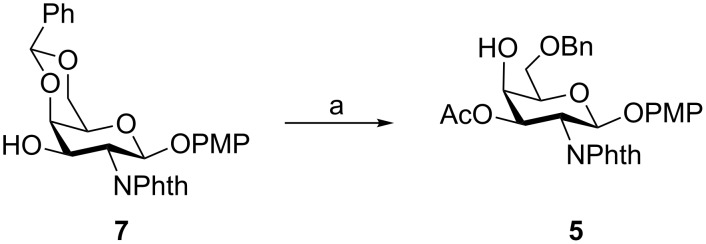
Reagents and conditions: (a) (i) acetic anhydride, pyridine, room temperature, 2 h; (ii) NaBH_3_CN, HCl/Et_2_O, 5 °C, 2 h, 77% overall yield.

Treatment of *p*-methoxyphenyl 4,6-*O*-benzylidene-2-deoxy-2-phthalimido-β-D-galactopyranoside (**7**) [24] (prepared from D-galactosamine hydrochloride in six steps) with acetic anhydride in pyridine followed by regioselective reductive opening of the benzylidene acetal on treatment with sodium cyanoborohydride in the presence of HCl/Et_2_O [25] furnished *p*-methoxyphenyl 3-*O*-acetyl-6-*O*-benzyl-2-deoxy-2-phthalimido-β-D-galactopyranoside (**5**) in 77% yield over two steps (Scheme 1).

**Scheme 2:**
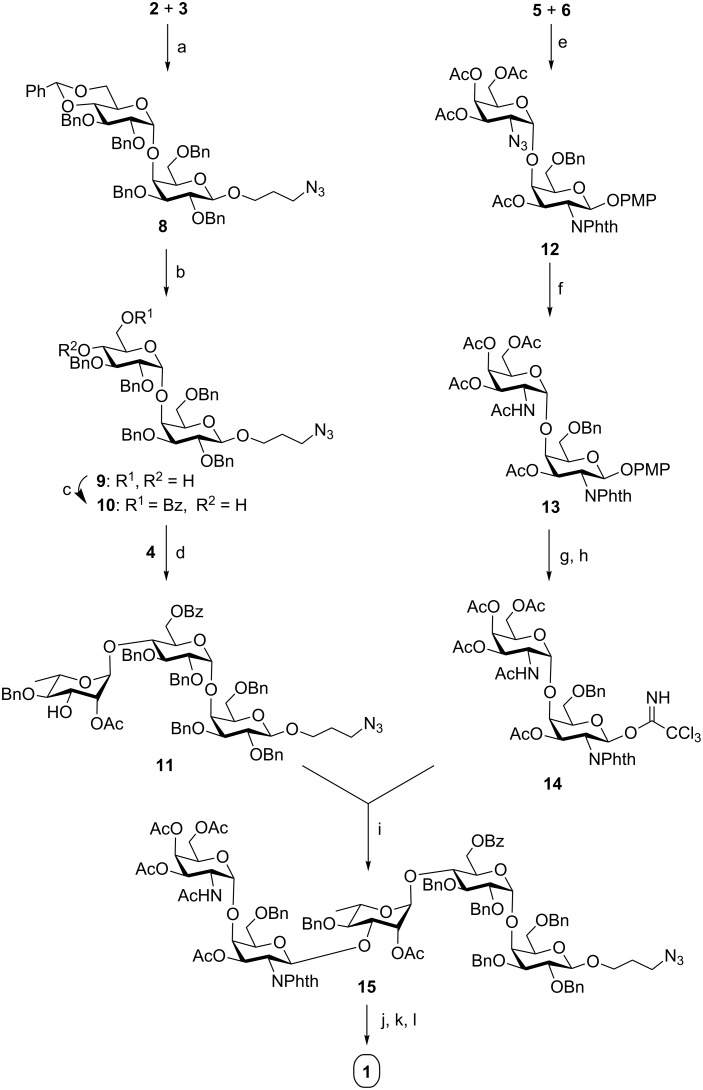
Reagents: (a) *N*-iodosuccinimide (NIS), TMSOTf, CH_2_Cl_2_, MS 4 Å, −30 °C, 1 h, 72%; (b) HClO_4_/SiO_2_, CH_3_CN, rt, 20 min, 85%; (c) benzoyl cyanide, DCM/pyridine, rt, 2 h, 80%; (d) *N*-iodosuccinimide (NIS), TfOH, CH_2_Cl_2_, MS 4 Å, −30 °C, 1 h, then 0 °C, 1 h, 77%; (e) NOBF_4_, Et_2_O/CH_2_Cl_2_ (3:1), −15 °C, 1 h, 75%; (f) PPh_3_, THF, 6 h, then Ac_2_O, pyridine, rt, 1 h, 84%;(g) CAN, CH_3_CN, H_2_O, rt, 1.5 h; (h) CCl_3_CN, DBU, CH_2_Cl_2_, −10 °C, 1 h; (i) NOBF_4_, CH_2_Cl_2_, −15 °C, 1 h, 70%; (j) i) NH_2_NH_2_·H_2_O, CH_3_CH_2_OH, 80 °C, 8 h, ii) acetic anhydride, pyridine, rt, 1 h; (k) H_2_, 20% Pd(OH)_2_/C, CH_3_OH, rt, 24 h; (l) 0.1 M CH_3_ONa, CH_3_OH, rt, 3 h, 58% overall yield.

Trisaccharide acceptor **11** could be synthesized following the reaction pathway depicted in Scheme 2. Glycosylation of 3-azidopropyl 2,3,6-tri-*O*-benzyl-β-D-galactopyranoside (**2**) with the thioglycoside donor **3** in the presence of *N*-iodosuccinimide (NIS) and trimethylsilyl trifluoromethanesulfonate (TMSOTf) [26–27] gave disaccharide derivative **8** in 72% yield. NMR spectroscopy confirmed the formation of compound **8** [δ 5.04 (d, *J* = 3.6 Hz, 1H, H-1_B_), 4.38 (d, *J* = 7.6 Hz, 1H, H-1_A_) in ^1^H NMR and at δ 103.9 (C-1_A_), 100.5 (C-1_B_) in ^13^C NMR spectra]. Following an earlier report [28], cleavage of the benzylidene acetal from compound **8** catalyzed by perchloric acid on silica (HClO_4_/SiO_2_) [28–29] afforded 3-azidopropyl (2,3-di-*O*-benzyl-β-D-glucopyranosyl)-(1→4)-2,3,6-tri-*O*-benzyl-β-D-galactopyranoside (**9**) in 85% yield. Selective 6-*O*-benzoylation of compound **9** was accomplished with benzoyl cyanide [30] to furnish disaccharide acceptor **10** in 80% yield. Compound **10** was reacted with 3-*O*-PMB protected L-rhamnosylthioglycoside donor **4** and NIS/TfOH [26–27] to yield the trisaccharide derivative by an iodonium ion catalyzed glycosylation. Participation of the 2-*O*-acetyl group in donor **4** ensured the α-selectivity of the glycosylation. Following an earlier report [19], raising the temperature of the reaction mixture after the glycosylation led to the removal of the PMB group in the same pot [31] to furnish trisaccharide acceptor **11** in 77% yield. The formation of compound **11** was supported by NMR spectral analysis [signals at δ 4.85 (d, *J* = 2.4 Hz, 1H, H-1_B_), 4.84 (brs, 1H, H-1_C_), 4.18 (d, *J* = 7.4 Hz, 1H, H-1_A_) and at δ 104.1 (C-1_A_), 98.7 (C-1_B_), 97.6 (C-1_C_) in the ^1^H and ^13^C NMR spectra respectively]. In another experiment, coupling of 2-azido-α-D-galactosyl trichloroacetimidate derivative **6** and compound **5** in the presence of NOBF_4_ [32] in Et_2_O/CH_2_Cl_2_ gave disaccharide derivative **12** in 75% yield. The formation of compound **12** was confirmed by NMR spectroscopic analysis [signals at δ 5.92 (d, *J* = 8.1 Hz, 1H, H-1_D_), 5.06 (d, *J* = 2.7 Hz, 1H, H-1_E_) in the ^1^H NMR and δ 99.1 (C-1_E_), 97.7 (C-1_D_) in the ^13^C NMR, respectively]. Reduction of the azido groups were carried out by treatment with triphenylphosphine [33], then the product was acetylated using acetic anhydride and pyridine to give disaccharide derivative **13** in 84% overall yield in two steps. The anomeric PMP group of compound **13** was oxidatively cleaved using ceric(IV) ammonium nitrate (CAN) [15] and the hemeacetal thus obtained was reacted with trichloroacetonitrile in the presence of DBU [34] to afford the desired disaccharide trichloroacetimidate derivative **14** in 77% yield. It was used directly without further purification [17] (Scheme 2). Finally, glycosylation of trisaccharide acceptor **11** with the trichloroacetimidate donor **14** in the presence of NOBF_4_ [32] in CH_2_Cl_2_ furnished pentasaccharide derivative **15** in 70% yield. Formation of compound **15** was supported by NMR spectral analysis [signals at δ 5.10 (d, *J* = 3.3 Hz, 1H, H-1_E_), 5.00 (d, *J* = 2.4 Hz, 1H, H-1_B_), 4.92 (brs, 1H, H-1_C_), 4.69 (d, *J* = 7.7 Hz, 1H, H-1_D_),4.35 (d, *J* = 7.8 Hz, 1H, H-1_A_) and at δ 104.0 (C-1_A_), 101.2 (C-1_D_), 98.9 (C-1_B_), 98.3 (C-1_C_), 97.4 (C-1_E_) in the ^1^H and ^13^C NMR spectra, respectively]. The *N*-phthaloyl group was removed using hydrazine hydrate and the free amine thus formed was acetylated using acetic anhydride in pyridine [35]. Then the removal of the benzyl group was accomplished by hydrogenation using Pearlman’s catalyst [36]. Finally the acetates were removed by Zemplén de-O-acetylation [37] using sodium methoxide to afford the target pentasaccharide **1** in 58% overall yield (Scheme 2).

## Conclusion

In summary, a [3 + 2] block glycosylation strategy has been developed to synthesize a pentasaccharide 3-aminopropyl glycoside (**1**) corresponding to the *O*-antigen of *E. coli* O117:K98:H4 strain. The in situ removal of the PMB ether in one-pot following the glycosylation reaction reduced the overall number of steps.

## Supporting Information

File 1 Experimental part.
