# Supplementary material for: Synthesis of the pentasaccharide repeating unit of the O-antigen of E. coli O117:K98:H4
Source: Beilstein J Org Chem. 2014 Nov 20;10:2724–8. doi: 10.3762/bjoc.10.287 (PMC4273275; doi:10.3762/bjoc.10.287)
Supplement: File 1 — Experimental part. [file Beilstein_J_Org_Chem-10-2724-s001.pdf]

Supporting Information

for

Synthesis of the pentasaccharide repeating unit of the *O*-antigen of *E. coli* O117:K98:H4

Pintu Kumar Mandal*

Address: Medicinal and Process Chemistry Division, CSIR-Central Drug Research Institute, BS-10/1, Sector 10, Jankipuram extension, Sitapur Road, Lucknow, 226 031, India.

* Corresponding author

Email: Pintu Kumar Mandal* - *pintuchem06@gmail.com*

Experimental part

Table of Contents **Page No.**

Experimental procedure **S2–S11**

1D and 2D NMR spectra of compounds **1, 5, 8, 9, 10, 11, 12, 13, 15** **S12–S28**

General methods

All reactions were monitored by thin-layer chromatography over silica gel-coated TLC plates. The spots on TLC were visualized by warming ceric sulfate [2% $Ce(SO_4)_2$ in 5% H_2SO_4 in EtOH]-sprayed plates on a hot plate. Silica gel 230–400 mesh was used for column chromatography. ^1H and ^{13}C NMR, DEPT 135, 2D COSY, HSQC spectra were recorded on Brucker DPX 400 MHz spectrometers using $CDCl_3$and D_2O as solvents and TMS as internal reference unless stated otherwise. Chemical shift values are expressed in δ ppm. ESI-MS were recorded on a JEOL spectrometer. Elementary analysis was carried out on Carlo ERBA analyzer. IR spectra were recorded on Shimadzu Spectrophotometers. Optical rotations were determined on Autopol III polarimeter. Commercially available grades of organic solvents of adequate purity are used in all reactions.

***p*-Methoxyphenyl 3-*O*-acetyl-6-*O*-benzyl-2-deoxy-2-phthalimido-β-D-galactopyranoside (5):** A solution of compound **7** (3 g, 5.96 mmol) in acetic anhydride (6 mL) and pyridine (6 mL) was allowed to stir at room temperature for 2 h. The solvents were removed under reduced pressure to give the acetylated product. A solution of the acetylated product in anhydrous THF (50 mL) were added $NaBH_3CN$ (0.75 g, 11.92 mmol) and MS 3 Å (1 g) and the reaction mixture was allowed to stir at 0 °C for 15 min. To the cooled reaction mixture was added dropwise HCl/Et_2O (~10 mL) until the pH of the solution became ~2. After stirring the reaction mixture at 5 °C for 2 h, it was poured into a satd. $NaHCO_3$ solution and extracted with CH_2Cl_2 (150 mL). The organic layer was washed with water, dried (Na_2SO_4), and concentrated to give the crude product, which was purified over SiO_2 using hexane/ EtOAc (3:1) as eluant to give pure compound **5** (2.5 g, 77%). Colorless oil; $[\alpha]_D^{25}$+10 (*c* 1.0, $CHCl_3$); IR (neat): 3064, 2865, 1772, 1710, 1609, 1507, 1090, 880 cm^{-1}; ^1H NMR (400 MHz, $CDCl_3$): δ7.85 (brs, 2 H, Ar-H), 7.78-7.72 (m, 2 H, Ar-H), 7.36-7.28 (m, 5 H, Ar-H), 6.87 (d, J = 9.1 Hz, 2 H, Ar-H), 6.70 (d, J = 9.1 Hz, 2 H, Ar-H), 5.81(d, J = 8.5 Hz, 1 H, H-1_D), 5.71

(dd, J = 11.1, 3.3 Hz, 1 H, H-3$_D$), 4.87 (dd, J = 11.2, 8.5 Hz, 1 H, H-2$_D$), 4.59 (ABq, 2 H, PhCH_2), 4.31 (brs, 1 H, H-5$_D$), 3.98-3.95 (m, 1 H. H-4$_D$), 3.88-3.80 (m, 2 H, H-6$_{abD}$), 3.71 (O**C**H_3), 1.97 (s, 3 H, COCH_3); ^{13}C NMR (100 MHz, CDCl$_3$): δ169.9 (*C*OCH$_3$), 168.0, 167.7 (*C*OPhth), 155.5-114.4 (Ar-C), 97.7 (C-1$_D$), 73.8 (C-5$_D$), 73.4 (C-3$_D$), 70.6 (Ph*C*H$_2$), 69.5 (C-6$_D$), 67.5 (C-4$_D$), 55.6 (O*C*H$_3$), 51.2 (C-2$_D$), 20.8 (CO*C*H$_3$); ESI-MS: *m/z* 570.1 [M+Na]$^+$; Anal. Calcd.for $C_{30}H_{29}NO_9$ (547.18): C, 65.81; H 5.34%; found C, 65.67; H, 5.48%.

3-Azidopropyl (2,3-di-*O*-benzyl-4,6-*O*-benzylidene-β-D-glucopyranosyl)-(1→4)-2,3,6-tri-*O*-benzyl-β-D-galactopyranoside (8): To a solution of compound **2** (5 g, 9.38 mmol) and compound **3** (5.54 g, 11.251 mmol) in anhydrous CH_2Cl_2 (80.0 mL) was added MS 4 Å (3.0 g) and the reaction mixture was stirred under argon at room temperature for 30 min and cooled to −30 °C. To the cooled reaction mixture were added NIS (3.04 g, 13.50 mmol) and TMSOTf (50μL) and it was stirred at same temperature for 1 h. The reaction mixture was filtered through a Celite® bed and washed with CH_2Cl_2 (200 mL). The combined organic layer was successively washed with 5% $Na_2S_2O_3$, satd. $NaHCO_3$ and water, dried (Na_2SO_4) and concentrated. The crude product was purified over SiO_2 using hexane/EtOAc (5:1) as eluant to give pure compound **8** (6.5g, 72%). Colorless oil; $[\alpha]_D^{25}$–27 (*c* 1.0, CHCl$_3$); IR (neat): 3725, 3417, 2144, 1742, 1666, 1629, 1517, 1061, 741cm^{-1}; ^{1}H NMR (400 MHz, CDCl$_3$): δ7.56-7.24 (m, 30 H, Ar-H), 5.57 (s, 1 H, PhC*H*), 5.04 (d, J = 3.6 Hz, 1 H, H-1$_B$), 5.03 (d, J = 11.1 Hz, 1 H, PhCH_2), 4.96 (d, J = 11.8 Hz, 1 H, PhCH_2), 4.88 (dd, J = 11.8 Hz, 2 H, PhCH_2), 4.82(d, J = 11.7 Hz, 1 H, PhCH_2), 4.72 (dd, J = 11.8 Hz, 2 H, PhCH_2), 4.62 (d, J = 11.8 Hz, 1 H, PhCH_2), 4.51 (d, J = 11.9 Hz, 1 H, PhCH_2), 4.38 (d, J = 7.6 Hz, 1 H, H-1$_A$), 4.34 (brs, 1 H, PhCH_2), 4.33-4.29 (m, 1 H, H-4$_B$), 4.24 (dd, J = 9.9, 8.4 Hz, 1 H, H-2$_A$), 4.09 (d, J = 2.8 Hz, 1 H, H-4$_A$), 4.07-3.98 (m, 2 H, H-6$_{aB}$,-OCH_2-), 3.93 (dd, J =9.8, 7.8 Hz, 1 H, H-6$_{aA}$), 3.78 (dd, J = 10.2, 9.9 Hz, 1H, H-3$_B$), 3.69-3.63 (m, 3 H, H-3$_A$,H-5$_B$, -OCH_2-), 3.62-3.54 (m, 3 H, H-2$_B$, H-6$_{bA}$, H-6$_{bB}$), 3.49-3.46 (m, 1 H, H-5$_A$), 3.44 (t, J = 6.8 Hz, 2 H,

CH_2N_3), 1.98-1.87 (m, 2 H, -CH_2-); ^{13}C NMR (100 MHz, $CDCl_3$): δ137.7-126.0 (Ar-C), 103.9 (C-1_A), 101.1 (Ph*C*H), 100.5 (C-1_B), 82.9 (C-5_B), 80.6 (C-5_A), 79.7 (C-3_A), 78.8 (C-3_B), 78.7 (C-2_A), 75.6 (C-4_A), 75.1 (PhCH_2), 75.0 (PhCH_2), 73.9 (PhCH_2), 73.4 (C-2_B), 73.1 (PhCH_2), 72.7 (PhCH_2), 69.0 (C-6_A), 67.9 (C-6_B), 66.5 (-OCH_2-), 62.9 (C-4_B), 48.3 (CH_2N_3), 29.3 (-CH_2-); ESI-MS: *m/z*986.2 $[M+Na]^+$; Anal. Calcd.for $C_{57}H_{61}N_3O_{11}$ (963.43): C, 71.01;H6.38%; found C, 70.91; H, 6.51%.

3-Azidopropyl (2,3-di-*O*-benzyl-β-D-glucopyranosyl)-(1→4)-2,3,6-tri-*O*-benzyl-β-D-galactopyranoside (9): To a solution of compound **8** (4 g, 4.15 mmol) in CH_3CN (50 mL) was added $HClO_4/SiO_2$ (0.5 g) and the reaction mixture was allowed to stir at room temperature for 20 min. The reaction mixture was filtered and the filtrate was evaporated to dryness. The crude product was purified over SiO_2 using hexane/EtOAc (2:1) to give pure compound **9** (3.08 g, 85%); $[\alpha]_D^{25}$–22 (*c* 1.0, $CHCl_3$); IR (neat): 3407, 2917, 2324, 1536, 1425, 1352, 1216, 1161, 1048, 742, 696 cm^{-1}; ^{1}H NMR (400 MHz, $CDCl_3$): δ7.43-7.25 (m, 25 H, Ar-H), 5.02 (d, *J* = 11.6 Hz, 1 H, PhCH_2), 5.01 (d, *J* = 2.8 Hz, 1 H, H-1_B), 4.91 (d, *J* = 11.9 Hz, 1 H, PhCH_2), 4.82 (dd, *J* = 11.9 Hz, 4 H, PhCH_2), 4.72 (dd, *J* = 12.0 Hz, 2 H, PhCH_2), 4.39 (d, *J* = 7.6 Hz, 1 H, H-1_A), 4.36 (brs, 2 H, PhCH_2), 4.08-4.01 (m, 3 H, H-2_B, H-3_B, -OCH_2-), 3.98-3.94 (m, 2 H, H-3_A, H-6_{aB}), 3.76 (dd, *J* = 9.8, 7.5 Hz, 1 H, H-2_A), 3.72-3.64 (m, 2 H, H-6_{bB}, -OCH_2-), 3.61-3.51 (m, 5 H, H-4_A, H-4_B, H-5_B,H-6_{abA}), 3.48-3.47 (m, 1 H, H-5_A), 3.46 (t, *J* = 6.7 Hz, 2 H, CH_2N_3), 1.97-1.87 (m, 2 H, -CH_2-); ^{13}C NMR (100 MHz, $CDCl_3$): δ138.8-127.6 (Ar-C), 103.9 (C-1_A), 99.6 (C-1_B), 81.3(C-3_B), 80.8 (C-5_A), 80.4 (C-4_A), 79.0 (C-2_A), 76.0 (C-3_A), 75.3 (Ph*C*H2), 75.2 (PhCH_2), 73.6 (C-5_B), 73.4 (PhCH_2), 73.2 (PhCH_2), 72.8 (PhCH_2), 71.8 (C-2_B), 70.9 (C-4_B), 68.4 (C-6_B), 66.6 (-OCH_2-), 62.2 (C-6_A), 48.3 (CH_2N_3), 29.3 (-CH_2-); ESI-MS: *m/z* 898.2 $[M+Na]^+$; Anal. Calcd.for $C_{50}H_{57}N_3O_{11}$ (875.40): C, 68.55; H, 6.56%; found C, 68.41; H, 6.71%.

3-Azidopropyl (6-*O*-benzoyl-2,3-di-*O*-benzyl-β-D-glucopyranosyl)-(1→4)-2,3,6-tri-*O*-benzyl-β-D-galactopyranoside (10): To a solution of compound **9** (3 g, 3.43 mmol) in CH_2Cl_2 (30 mL) were added pyridine (5 mL) and benzoyl cyanide (0.49 g, 3.77 mmol) and the reaction mixture was stirred at room temperature for 2 h. The reaction mixture was diluted with CH_2Cl_2 (100 mL) and successively washed with 1 M HCl, satd $NaHCO_3$ and water, dried (Na_2SO_4), and concentrated. The crude product was purified over SiO_2 using hexane/Et_2O (3:1) as eluant to give pure compound **10** (2.7g, 80%); $[\alpha]_D^{25}$ −15 (*c* 1.0, $CHCl_3$);IR (neat):2917, 2140, 1721, 1597, 1503, 1451, 1276, 1221, 1066, 750cm^{-1}; ^{1}H NMR (400 MHz, $CDCl_3$): δ7.93-7.91 (d, *J* = 8.2 Hz, 2 H, Ar-H), 7.41-7.08 (m, 28 H, Ar-H), 4.96 (d, *J* = 2.9 Hz, 1 H, H-1_B), 4.82 (dd, *J* = 11.9 Hz, 3 H, PhC*H*$_2$), 4.73 (d, *J* = 12.0 Hz, 1 H, PhC*H*$_2$), 4.69 (d, *J* = 11.8 Hz, 1 H, PhC*H*$_2$), 4.68 (d, *J* = 11.8 Hz, 1 H, PhC*H*$_2$), 4.59 (dd, *J* = 11.8 Hz, 2 H, PhC*H*$_2$), 4.39 (dd, *J* = 12.3, 1.3 Hz, 1H, H-6_{aA}), 4.22 (d, *J* = 7.7 Hz, 1 H, H-1_A), 4.21-4.19 (m, 3 H, H-2_B, PhC*H*$_2$), 3.99 (d, *J* = 2.1 Hz, 1 H, H-4_A), 3.92-3.86 (m, 4 H, H-2_A, H-6_{bA}, H-6_{aB}, -OC*H*$_2$-), 3.63 (dd, *J* = 9.8, 7.5 Hz, 1 H, H-3_A), 3.56-3.52 (m, 1 H, -OC*H*$_2$-), 3.51-3.39 (m, 4 H, H-3_B,H-4_B, H-5_B, H-6_{bB}), 3.36-3.33 (m, 1 H, H-5_A), 3.30 (t, *J* = 6.7 Hz, 2 H, C*H*$_2$$N_3$), 1.82-1.77 (m, 2 H, -C*H*$_2$-); ^{13}C NMR (100 MHz, $CDCl_3$): δ167.2 (*C*OPh), 138.7-127.6 (Ar-C), 103.9 (C-1_A), 99.6 (C-1_B), 80.9 (C-2_A), 80.5 (C-5_A), 80.2 (C-3_B), 78.9 (C-3_A), 75.5 (C-4_A), 75.4 (Ph*C*H$_2$), 75.2 (Ph*C*H$_2$), 73.5 (C-5_B), 73.4 (Ph*C*H$_2$), 73.1 (Ph*C*H$_2$), 72.8 (Ph*C*H$_2$), 70.6 (C-2_B), 70.4 (C-4_B), 67.9 (C-6_B), 66.6 (-O*C*H$_2$-), 63.2 (C-6_A), 48.4 (*C*H$_2$$N_3$), 29.3 (-*C*H$_2$-); ESI-MS: *m/z*1002.2 [M+Na]$^+$; Anal. Calcd.for $C_{57}H_{61}N_3O_{12}$ (979.43): C, 69.85; H, 6.27%; found C, 69.71; H, 6.41%.

3-Azidopropyl (2-*O*-acetyl-4-*O*-benzyl-α-L-rhamnopyranosyl)-(1→4)-(6-*O*-benzoyl-2,3-di-*O*-benzyl-β-D-glucopyranosyl)-(1→4)-2,3,6-tri-*O*-benzyl-β-D-galactopyranoside (11): To a solution of compound **10** (2 g, 2.04 mmol) and compound **4** (1.1 g, 2.45 mmol) in anhydrous CH_2Cl_2 (10 mL) was added MS 4 Å (2 g) and the reaction mixture was stirred

under argon at room temperature for 30 min and cooled to −30 °C. To the cooled reaction mixture were added NIS (0.66 g, 2.94 mmol) and TfOH (30 μL) and it was stirred at the same temperature for 1 h. The temperature of reaction mixture was raised to 0 °C and it was stirred at 0 °C for another 1 h. The reaction mixture was filtered through a Celite® bed and washed with CH_2Cl_2 (100 mL). The combined organic layer was successively washed with 5% $Na_2S_2O_3$, satd. $NaHCO_3$ and water, dried (Na_2SO_4) and concentrated. The crude product was purified over SiO_2 using hexane/EtOAc (4:1) as eluant to give pure compound **11** (1.97 g, 77%). Yellow oil;$[\alpha]_D^{25}$ +23 (*c* 1.0, $CHCl_3$);IR (neat): 3418, 2911, 2769, 2141, 1591, 1354, 1066, 762cm^{-1}; ^{1}H NMR (400 MHz, $CDCl_3$): δ7.87 (d, *J* = 8.2 Hz, 2 H, Ar-H), 7.23-6.99 (m, 33 H, Ar-H), 4.85 (d, *J* = 2.4 Hz, 1 H, H-1_B), 4.84 (brs, 1 H, H-1_C), 4.83 (brs, 2 H, H-2_C, PhCH_2), 4.72 (dd, *J* = 11.8 Hz, 2 H, PhCH_2), 4.62 (dd, J = 11.9 Hz, 4 H, PhCH_2), 4.51 (d, *J* = 12.0 Hz, 1 H, PhCH_2), 4.48 (dd, *J* = 11.2 Hz, 2 H, PhCH_2), 4.30 (dd, *J* = 12.3, 1.3 Hz, 1 H, H-6_{aA}), 4.18 (d, *J* = 7.4 Hz, 1 H, H-1_A), 4.17-4.14 (m, 1 H, H-2_B), 4.10 (brs, 2 H, PhCH_2), 3.97-3.93 (m, 1 H, H-3_C), 3.87-3.81 (m, 5 H, H-3_B, H-4_A, H-5_C, H-6_{bA},-OCH_2-), 3.73-3.69 (m, 2 H, H-4_B, H-6_{aB}), 3.58-3.47 (m, 2 H, H-3_A, -OCH_2-), 3.39-3.32 (m, 3 H, H-2_A, H-5_B,H-6_{bB}), 3.30-3.27 (m, 1 H, H-5_A), 3.25 (t, *J* = 6.7 Hz, 2 H, CH_2N_3), 3.13 (t, *J* = 9.4 Hz, 1 H, H-4_C), 1.86 (s, 3 H, COCH_3), 1.81-1.72 (m, 2 H, -CH_2-), 0.91 (d, *J* = 6.1 Hz, 3 H, CCH_3); ^{13}C NMR (100 MHz, $CDCl_3$): δ170.6 (*C*OCH$_3$), 166.0 (*C*OPh), 104.1 (C-1_A), 98.7 (C-1_B), 97.6 (C-1_C), 81.7 (C-4_C), 81.2 (C-2_A), 80.3 (C-5_A), 79.5 (C-3_B), 78.9 (C-3_A), 75.6 (C-4_A), 75.4 (Ph*C*H$_2$), 75.2 (C-4_B), 75.1 (Ph*C*H$_2$), 75.0 (Ph*C*H$_2$), 73.6 (Ph*C*H$_2$), 73.5 (C-5_B), 73.2 (Ph*C*H$_2$), 72.9 (Ph*C*H$_2$), 72.8 (C-2_C), 69.9 (C-3_C), 69.5 (C-2_B), 68.2 (C-5_C), 68.0 (C-6_B), 66.7 (-O*C*H$_2$-), 62.7 (C-6_A), 48.4 (*C*H$_2$N$_3$), 29.3 (-*C*H$_2$-), 20.9 (CO*C*H$_3$), 17.9 (C*C*H$_3$); ESI-MS: *m/z*1280.3 [M+Na]$^+$; Anal. Calcd.for $C_{72}H_{79}N_3O_{17}$ (1257.54): C, 68.72; H, 6.33%; found C, 68.58; H, 6.45%.

***p*-Methoxyphenyl (3,4,6-tri-*O*-acetyl-2-azido-2-deoxy-α-D-galactopyranosyl)-(1→4)-(3-*O*-acetyl-6-*O*-benzyl-2-deoxy-2-phthalimido-β-D-galactopyranoside (12):** To a solution of compound **5** (2 g, 3.66 mmol) and trichloroacetimidate donor **6** (2.08 g, 4.39 mmol) in dry Et_2O/CH_2Cl_2 (50 mL; 3:1; v/v) was cooled to −15 °C. To the cooled reaction mixture was added $NOBF_4$ (400 mg, 3.42 mmol) and the reaction mixture was allowed to stir at the same temperature for 1 h. When TLC showed complete consumption of the acceptor **5** then the reaction mixture was diluted with CH_2Cl_2 (100 mL) and the organic layer was washed with satd. $NaHCO_3$ and water, dried (Na_2SO_4) and evaporated to dryness. The crude product was purified over SiO_2 using hexane/EtOAc (5:1) as eluent to furnish pure compound **12** (2.35 g, 75%); yellow oil; $[\alpha]_D^{25}$ +18 (*c* 1.0, $CHCl_3$); IR (neat): 3429, 2927, 2369, 2111, 1752, 1717, 1635, 1574, 1377, 1228, 1070, 906cm^{-1}; ^{1}H NMR (400 MHz, $CDCl_3$): δ7.88 (brs, 2 H, Ar-H), 7.77-7.76 (m, 2 H, Ar-H), 7.74-7.31 (m, 5 H, Ar-H), 6.75 (d, *J* = 9.1 Hz, 2 H, Ar-H), 6.76 (d, *J* = 9.1 Hz, 2 H, Ar-H), 5.92 (d, *J* = 8.1 Hz, 1 H, H-1_D), 5.72 (dd, *J* = 9.2, 1.9 Hz, 1 H, H-3_D), 5.65 (dd, *J* = 2.2 Hz, 1 H, H-4_E), 5.53 (dd, *J* = 10.5, 2.6 Hz, 1 H, H-3_E), 5.06 (d, *J* = 2.7 Hz, 1 H, H-1_E), 4.84 (dd, *J* = 9.2, 6.8 Hz, 1 H, H-2_D), 4.72-4.69 (m, 1 H, H-4_D), 4.63-4.60 (m, 2 H, $PhCH_2$),4.41 (d, *J* = 2.2 Hz, 1 H, H-5_E), 4.22-4.13 (m, 2 H, H-6_{abE}), 4.09-4.03 (m, 3 H, H-5_D, H-6_{aD}), 3.91-3.87 (m, 1 H, H-2_E), 3.86-3.73 (m, 1 H, H-6_{bD}), 3.74 (s, 3 H, OCH_3), 2.20 (s, 3 H, $COCH_3$), 2.13 (s, 3 H, $COCH_3$), 2.09 (s, 3 H, $COCH_3$), 1.94 (s, 3 H, $COCH_3$); ^{13}C NMR (100 MHz, $CDCl_3$): δ170.4 ($COCH_3$), 170.1, ($COCH_3$), 170.0 ($COCH_3$), 169.6 ($COCH_3$), 168.0, 167.7 (*C*OPhth), 155.5-114.2 (Ar-C), 99.1 (C-1_E), 97.7 (C-1_D), 74.9 (C-5_E), 73.6 (C-5_D), 73.4 ($PhCH_2$), 70.2 (C-3_D), 68.9 (C-3_E), 67.4 (2 C, C-4E, C-6_D), 67.0(C-4_D), 60.8(C-6_E), 58.6 (C-2_E), 55.6 (OCH_3), 51.5 (C-2_D), 20.7 (4 C, $COCH_3$); ESI-MS: *m/z* 883.1 $[M+Na]^+$; Anal. Calcd.for $C_{42}H_{44}N_4O_{16}$ (860.28): C, 58.60; H, 5.15%; found C, 58.47; H, 5.33%.

***p*-Methoxyphenyl (2-acetamido-3,4,6-tri-*O*-acetyl-2-deoxy-α-D-galactopyranosyl)-(1→4)-(3-*O*-acetyl-6-*O*-benzyl-2-deoxy-2-phthalimido-β-D-galactopyranoside (13):** A solution of the azidodisaccharide **12** (2 g, 2.33 mmol) and PPh_3 (0.73 g, 2.79 mmol) in THF (20 mL) and trace amount of water was stirred to rt for 6 h. The solvent was evaporated and co-evaporated with toluene. A solution of the crude product in pyridine (5 mL) and acetic anhydride (5 mL) was kept at room temperature for 1 h. The solvents were evaporated and the crude product was purified over SiO_2 usinghexane-EtOAc (5:1) as eluent to furnish pure compound **13** (1.7 g, 84%); yellow oil; $[\alpha]_D^{25}$ +22 (*c* 1.0, $CHCl_3$);IR (neat): 3057, 2990, 2829, 1695, 1602, 1508, 1466, 1369, 1341, 1170, 1125, 1055, 992, 857, 746cm^{-1}; ^{1}H NMR (400 MHz, $CDCl_3$): δ 7.65 (brs, 1 H, Ar-H), 7.55-7.54 (m, 1 H, Ar-H), 7.47-7.45 (m, 2 H, Ar-H), 7.36-7.27 (m, 5 H, Ar-H), 6.92 (d, *J* = 9.1 Hz, 2 H, Ar-H), 6.76 (d, *J* = 9.1 Hz, 2 H, Ar-H), 5.45 (d, *J* = 2.3 Hz, 1 H, H-4_E), 5.33 (dd, *J* =9.2, 1.9 Hz, 1 H, H-3_D), 5.26 (dd, *J* = 11.3, 3.2 Hz, 1 H, H-3_E), 5.11 (d, *J* = 8.2 Hz, 1 H, H-1_D), 5.07 (d, *J* = 3.6 Hz, 1 H, H-1_E), 4.66-4.60 (m, 2 H, H-2_E, H-4_D), 4.59 (d, *J* = 11.8 Hz, 1 H, PhCH_2), 4.43 (d, *J* = 11.8 Hz, 1 H, PhCH_2), 4.33-4.29 (m, 1 H, H-2_D), 4.28 (d, *J* = 2.4 Hz, 1 H, H-5_E), 4.15-4.14 (m, 2 H, H-6_{abE}), 3.85-3.82 (m, 1 H, H-5_D), 3.74 (s, 3 H, OCH_3), 3.62-3.53 (m, 2 H, H-6_{abD}), 2.16 (s, 3 H, COCH_3), 2.09 (s, 3 H, COCH_3), 2.02 (s, 3 H, COCH_3), 1.99 (s, 3 H, COCH_3), 1.89 (s, 3 H, COCH_3); ^{13}C NMR (100 MHz, $CDCl_3$): δ170.7 (2 C, *C*OCH$_3$), 170.6, (*C*OCH$_3$), 170.4 (*C*OCH$_3$), 170.3(*C*OCH$_3$), 169.0, 168.8 (*C*OPhth), 155.5-114.5 (Ar-C), 100.9 (C-1_D), 97.8 (C-1_E), 73.4 (Ph*C*H$_2$), 73.1 (C-5_D), 71.5 (C-5_E), 71.2 (C-3_D), 68.1 (C-3_E), 66.9 (C-4_E), 66.8 (C-6_D), 66.6 (C-4_D), 61.2 (C-6_E), 55.6 (O*C*H$_3$), 51.9 (C-2_D), 47.6 (C-2_E), 23.1 (NHCO*C*H$_3$), 20.8 (2 C, CO*C*H$_3$), 20.7 (2 C, CO*C*H$_3$); ESI-MS: *m/z* 899.1 $[M+Na]^+$; Anal. Calcd.for $C_{44}H_{48}N_2O_{17}$ (876.29): C, 60.27; H, 5.52%; found C, 60.12; H, 5.65%.

3-Azidopropyl (2-acetamido-3,4,6-tri-*O*-acetyl-2-deoxy-α-D-galactopyranosyl)-(1→4)-(3-*O*-acetyl-6-*O*-benzyl-2-deoxy-2-phthalimido-β-D-galactopyranosyl)-(1→3)-(2-*O*-acetyl-4-*O*-benzyl-α-L-rhamnopyranosyl)-(1→4)-(6-*O*-benzoyl-2,3-di-*O*-benzyl-β-D-glucopyranosyl)-(1→4)-2,3,6-tri-*O*-benzyl-β-D-galactopyranoside (15): To a solution of compound **13** (1 g, 1.14 mmol) in CH_3CN/H_2O (20 mL, 4:1 v/v) was added ammonium cerium nitrate (CAN, 0.94 g, 1.71mmol) and the reaction mixture was allowed to stir at room temperature for 2 h. The reaction mixture was diluted with CH_2Cl_2 (50 mL) and the organic layer was washed with satd $NaHCO_3$ (50 mL) and water (50 mL), dried (Na_2SO_4), and evaporated to dryness to give the disaccharide hemiacetal. To a solution of the hemiacetal in anhydrous CH_2Cl_2 (20 mL) was added trichloroacetonitrile (0.16 mL, 1.69 mmol) and the reaction mixture was cooled to −10 °C. To the cooled reaction mixture was added DBU (0.2 mL, 1.3 mmol) and it was allowed to stir at −10 °C for 1 h. The reaction mixture was evaporated to dryness and the crude product was passed through a short pad of SiO_2 to furnish compound **14** (0.80 g, 77%). A solution of compound **11** (0.80 g, 0.64 mmol) andcompound **14** (0.70 g, 0.76 mmol) in anhydrous CH_2Cl_2 (15 mL) was cooled to −15 °C. To the cooled reaction mixture was added $NOBF_4$ (90 mg, 0.77 mmol) and it was allowed to stir at −15 °C for 1 h. The reaction mixture was diluted with CH_2Cl_2 (30 mL) and the organic layer was washed with satd $NaHCO_3$ (20 mL) and water (20 mL) in succession, dried (Na_2SO_4), and evaporated to dryness. The crude product was purified over SiO_2 using hexane/EtOAc (4:1) as eluent to give pure **15** (0.90 g, 70%); yellow oil; $[\alpha]_D^{25}$ +28 (*c* 1.0, $CHCl_3$);IR (neat): 3027, 2890, 2329, 2144, 1602, 1508, 1456, 1321, 1150, 1055, 992, 867 cm^{-1}; ^1H NMR (400 MHz, $CDCl_3$): δ 8.04-8.03 (m, 2 H, Ar-H), 7.39-7.17 (m, 42 H, Ar-H), 5.51 (dd, *J* = 11.3, 2.2 Hz, 1 H, H-3_E), 5.46 (d, *J* = 2.3 Hz, 1 H, H-4_E), 5.42 (brs, 1 H, H-2_C), 5.39 (dd, *J* =9.1, 1.8 Hz, 1 H, H-3_D), 5.10 (d, *J* = 3.3 Hz, 1 H, H-1_E), 5.00 (d, *J* = 2.4 Hz, 1 H, H-1_B), 4.99-4.97 (m, 1 H, PhC*H*$_2$), 4.92 (brs, 1 H, H-1_C), 4.90-4.80 (m, 4 H, PhC*H*$_2$), 4.77-

4.74 (m, 1 H, PhCH_2), 4.73-4.71 (d, J = 11.3 Hz, 1 H, PhCH_2), 4.69 (d, J = 7.7 Hz, 1 H, H-1_D),4.68-4.64 (m, 1 H, PhCH_2), 4.61-4.54 (m, 2 H, H-6_{aA}, PhCH_2), 4.45 (dd, J = 11.7 Hz, 2 H, PhCH_2), 4.40 (d, J = 11.7 Hz, 1 H, PhCH_2), 4.39-4.36 (m, 2 H, H-2_E, H-4_D), 4.35 (d, J = 7.8 Hz, 1 H, H-1_A), 4.33 (brs, 2 H, PhCH_2), 4.22-4.19 (m, 1 H, H-2_B), 4.13-4.03 (m, 7 H, H-2_D, H-3_B, H-3_C, H-5_C, H-5_E, H-6_{abE}), 4.00-3.95 (m, 3 H, H-4_A, H-6_{bA}, H-6_{aD}), 3.90-3.83 (m, 2 H, H-6_{aB}, -OCH_2-), 3.74 (dd, J = 9.6, 4.4 Hz, 1 H, H-3_A), 3.67-3.53 (m, 4 H, H-4_B, H-6_{bB}, H-6_{bD}, -OCH_2-), 3.51-3.48 (m, 4 H, H-2_A, H-4_C, H-5_A, H-5_B), 3.45-3.43 (m, 1 H, H-5_D), 3.41 (t, J = 6.6 Hz, 2 H, NCH_2), 2.15 (s, 3 H, NHCOCH_3), 2.06 (s, 9 H, COCH_3), 2.04 (s, 6 H, COCH_3), 1.99-1.88 (m, 2 H, -CH_2-), 1.27 (d, J = 6.1 Hz, 3 H, CCH_3); ^{13}C NMR (100 MHz, $CDCl_3$): δ170.3 (2 C, *C*OCH$_3$), 170.2 (*C*OCH$_3$), 169.9 (2 C, *C*OCH$_3$), 169.5 (*C*OCH$_3$), 169.1, 168.9 (*C*OPhth), 166.8 (*C*OPh), 161.1-126.9 (Ar-C), 104.0 (C-1_A), 101.2 (C-1_D), 98.9 (C-1_B), 98.3 (C-1_C), 97.4 (C-1_E), 81.6 (C-4_C), 81.2 (C-2_A), 80.2 (C-5_A), 79.2 (C-3_B), 78.9 (C-3_A), 75.8 (C-4_A), 75.3(Ph*C*H$_2$), 74.9 (Ph*C*H$_2$), 74.8 (Ph*C*H$_2$), 73.7 (Ph*C*H$_2$), 73.6(Ph*C*H$_2$), 73.1 (Ph*C*H$_2$), 72.9 (Ph*C*H$_2$), 72.7 (C-4_B), 72.4 (C-5_D),72.0 (C-5_E), 71.7 (C-5_B), 71.1 (C-2_C),69.7 (C-3_C), 69.3 (C-3_D), 68.7 (C-3_E), 68.3 (C-2_B), 68.2 (C-5_C), 67.9(C-6_B), 67.2 (-O*C*H$_2$-), 66.7 (C-4_E), 66.3 (C-6_D), 64.5(C-4_D), 62.3 (C-6_A), 60.7 (C-6_E), 57.9 (C-2_D), 48.4 (C-2_E), 48.2 (*C*H$_2$N$_3$), 29.2 (-*C*H$_2$-), 21.1 (NHCO*C*H$_3$), 20.7 (3 C, CO*C*H$_3$), 20.6 (2 C, CO*C*H$_3$),17.8 (C*C*H$_3$); ESI-MS: *m/z* 2032.5 $[M+Na]^+$; Anal. Calcd. for $C_{109}H_{119}N_5O_{32}$(2009.78): C, 65.10; H, 5.96; found: C,64.93; H, 6.09.

3-Aminopropyl (2-acetamido-2-deoxy-α-D-galactopyranosyl)-(1→4)-(2-acetamido-2-deoxy-β-D-galactopyranosyl)-(1→3)-(α-L-rhamnopyranosyl)-(1→4)-(β-D-glucopyranosyl)-(1→4)-β-D-galactopyranoside (1): To a solution of compound **15** (0.80 g, 0.39 mmol) in EtOH (20 mL) was added hydrazine hydrate (2 mL) and the mixture was allowed to stir at 80 °C for 8 h. The solvents were removed under reduced pressure and a solution of the crude mass in pyridine (5 mL) and acetic anhydride (5 mL) was kept at room

temperature for 1 h. The solvents were evaporated and co-evaporated with toluene under reduced pressure. To a solution of the acetylated product in CH_3OH (20 mL) was added 20% $Pd(OH)_2/C$ (200 mg) and the reaction mixture was stirred at room temperature under a positive pressure of H_2 for 24 h. The reaction mixture was filtered through a Celite® bed, the filtering bed was washed with CH_3OH (10 mL) and the combined filtrate was concentrated under reduced pressure. A solution of the hydrogenated product in 0.1 M CH_3ONa in CH_3OH (10 mL) was allowed to stir at room temperature for 3 h. The reaction mixture was neutralized with Dowex 50W-X8 (H^+) resin, filtered and concentrated. The crude product was passed through a Sephadex® LH-20 column using CH_3OH/H_2O (3:1) as eluant to give pure compound **1** (220 mg, 58%). White powder; $[\alpha]_D^{25}$ + 12 (*c* 1.0, H_2O); IR (KBr): 3456, 2935, 1622, 1356, 1236, 1165, 1067, 697 cm^{-1};^1H NMR (400 MHz, D_2O): δ 4.86 (brs, 1 H, H-1_E), 4.85 (brs, 1 H, H-1_B), 4.83 (brs, 1 H, H-1_C), 4.54 (d, *J* = 7.6 Hz, 1 H, H-1_A), 4.41 (d, *J* = 7.8 Hz, 1 H, H-1_D), 4.18-4.11 (m, 2 H, H-2_E,H-5_D), 4.06-3.94 (m, 4 H, H-2_D, H-4_A, H-$5_{C,}$ -OCH_2-), 3.86-3.82 (m, 4 H, H-3_B, H-3_C, H-4_E, H-6_{aB}), 3.77-3.67 (m, 10 H, H-2_C, H-3_E, H-6_{abA}, H-6_{bB}, H-6_{abD}, H-6_{abE}, -OCH_2-), 3.64-3.46 (m, 10 H, H-2_A, H-2_B, H-3_A, H-3_D, H-4_B, H-4_C, H-4_D, H-5_A, H-5_B, H-5_E), 3.13-310 (m, 2 H, NCH_2), 2.01 (s, 6 H, NHCOCH_3), 1.97-1.91 (m, 2 H, -CH_2-), 1.21 (d, *J* = 6.1 Hz, 3 H, CCH_3); ^{13}C NMR (100 MHz, D_2O): δ 175.1, 174.9 (NH*C*OCH$_3$), 105.0 (C-1_A), 103.4 (C-1_D), 101.2 (C-1_C), 101.1 (C-1_B), 100.3 (C-1_E), 80.7 (C-3_C), 77.6 (2 C, C-4_A, C-4_B), 75.7 (C-3_E), 75.5 (C-4_D), 73.1 (2 C, C-5_A, C-5_D), 72.7 (C-2_C), 72.6 (C-3_A), 71.9 (C-5_B), 71.6 (2 C, C-2_A, C-2_B), 71.4 (C-5_E), 71.3 (2 C, C-3_B ,C-3_D), 70.8 (C-4_C), 69.5 (C-5_C), 69.1 (C-4_E), 68.2 (-O*C*H$_2$-), 61.5 (2 C, C-6_D, C-6_E), 60.5 (C-6_B), 60.2 (C-6_A), 51.9 (C-2_D), 49.4 (C-2_E), 38.1 (N*C*H$_2$), 27.3 (-*C*H$_2$-), 22.1 (2 C, NHCO*C*H$_3$), 17.1 (C*C*H$_3$); ESI-MS: *m/z* 952.2 $[M+H]^+$; Anal. Calcd. for $C_{37}H_{65}N_3O_{25}$(951.39): C, 46.68; H, 6.88; found: C,46.53; H, 6.99.

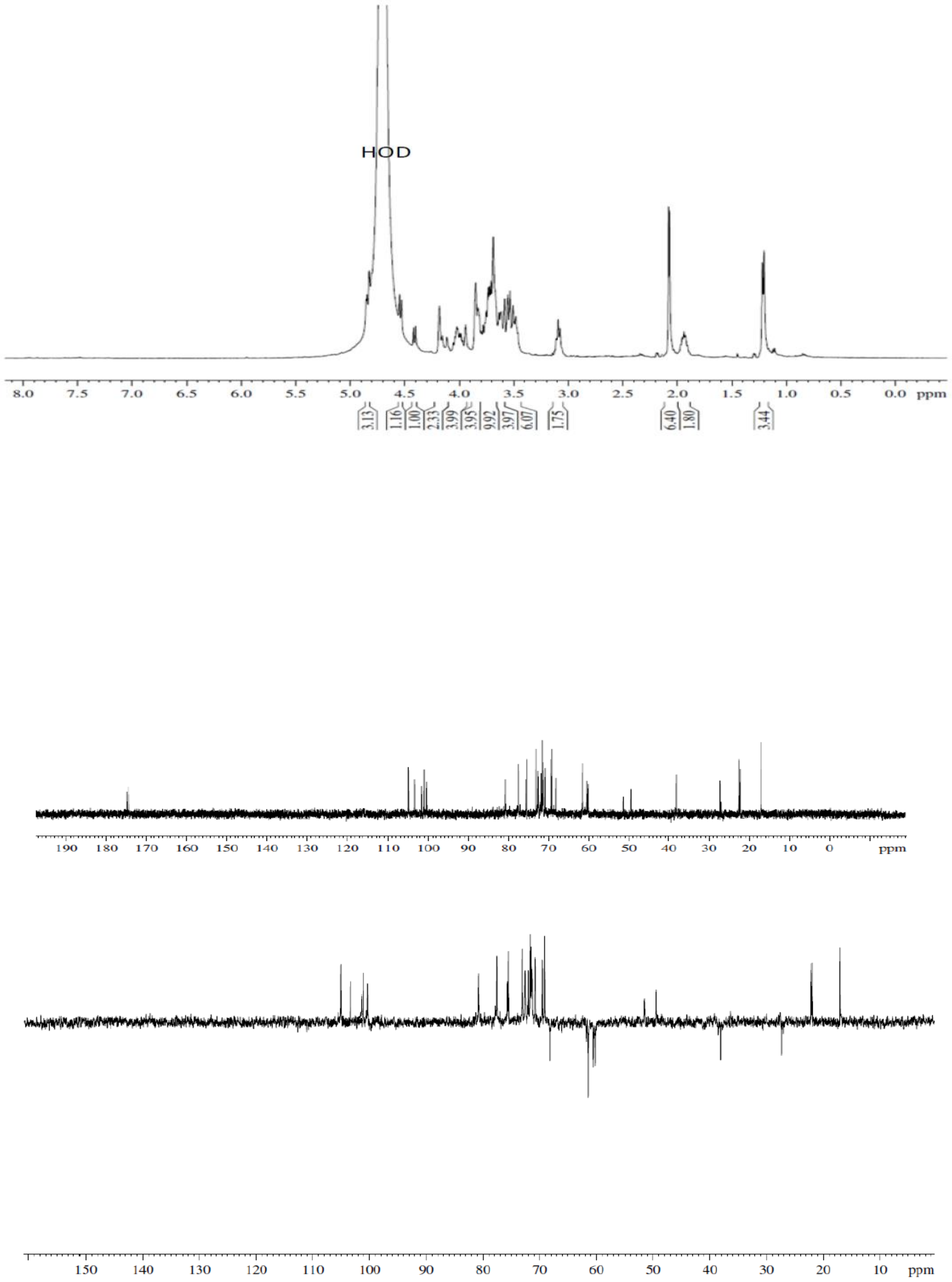

^{1}H, ^{13}C and DEPT 135 NMR spectra of 3-aminopropyl (2-acetamido-2-deoxy-α-D-galactopyranosyl)-(1→4)-(2-acetamido-2-deoxy-β-D-galactopyranosyl)-(1→3)-(α-L-rhamnopyranosyl)-(1→4)-(β-D-glucopyranosyl)-(1→4)-β-D-galactopyranoside (**1**) (D_2O).

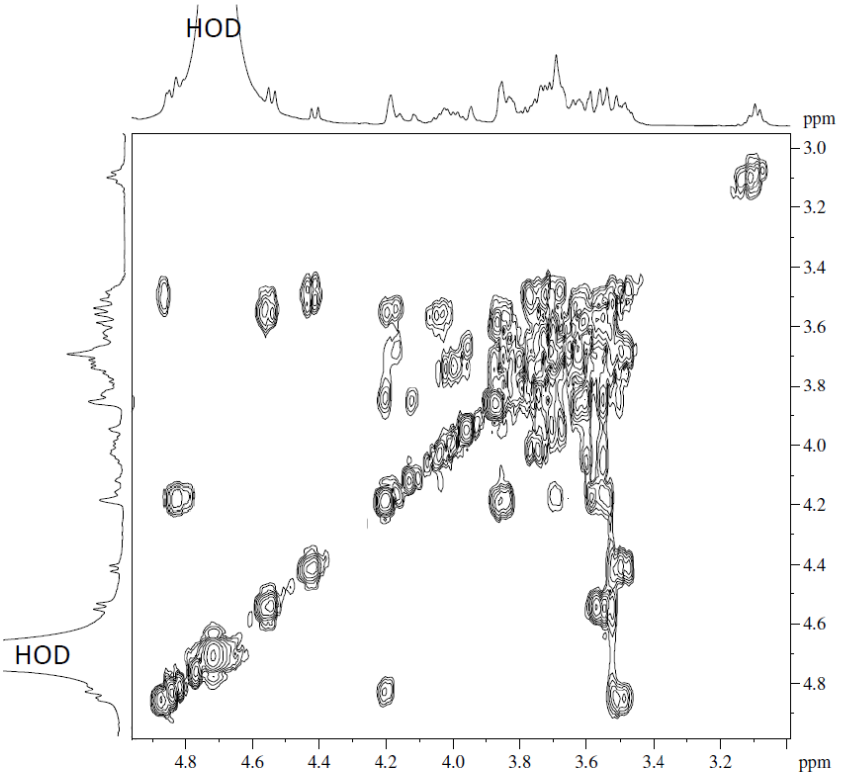

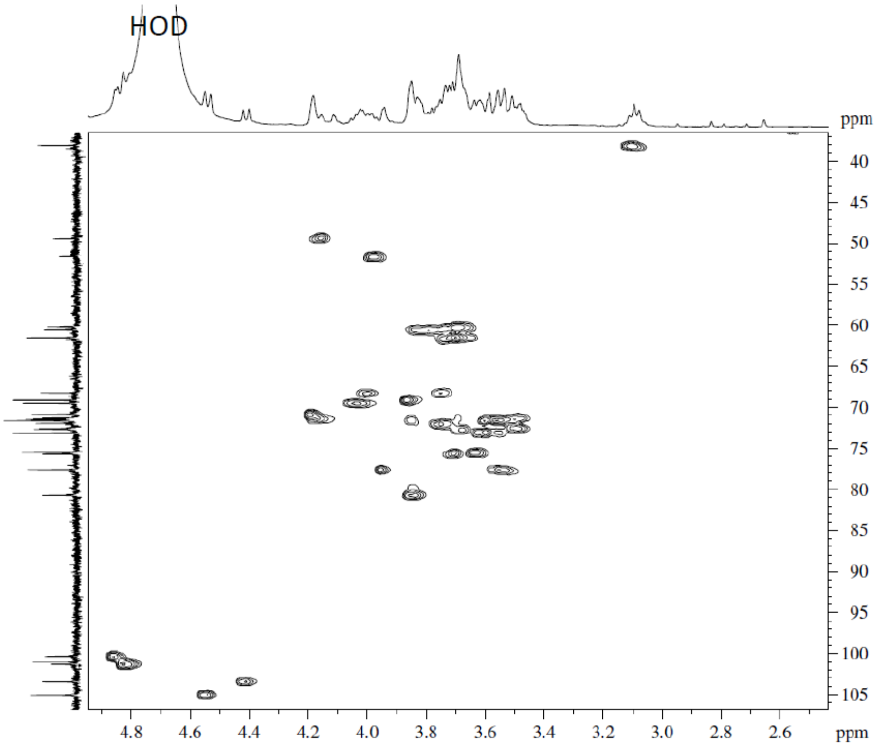

2D COSY and HSQC NMR spectra (selected regions) of 3-aminopropyl (2-acetamido-2-deoxy-α-D-galactopyranosyl)-(1→4)-(2-acetamido-2-deoxy-β-D-galactopyranosyl)-(1→3)-(α-L-rhamnopyranosyl)-(1→4)-(β-D-glucopyranosyl)-(1→4)-β-D-galactopyranoside (**1**) (D_2O).

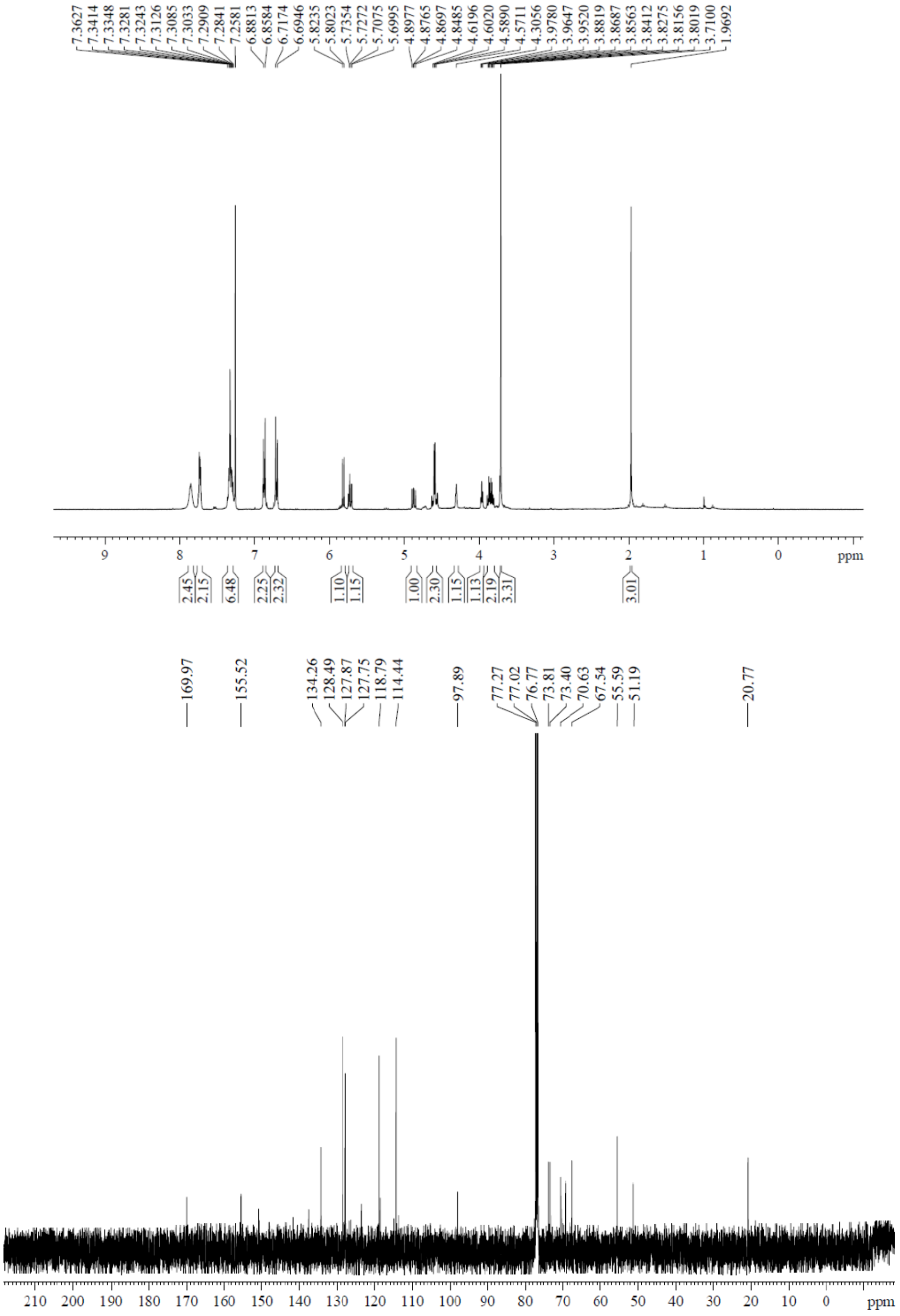

^{1}H, ^{13}C and DEPT 135 NMR spectra of *p*-methoxyphenyl 3-*O*-acetyl-6-*O*-benzyl-2-deoxy-2-phthalimido-β-D-galactopyranoside (**5**) ($CDCl_3$).

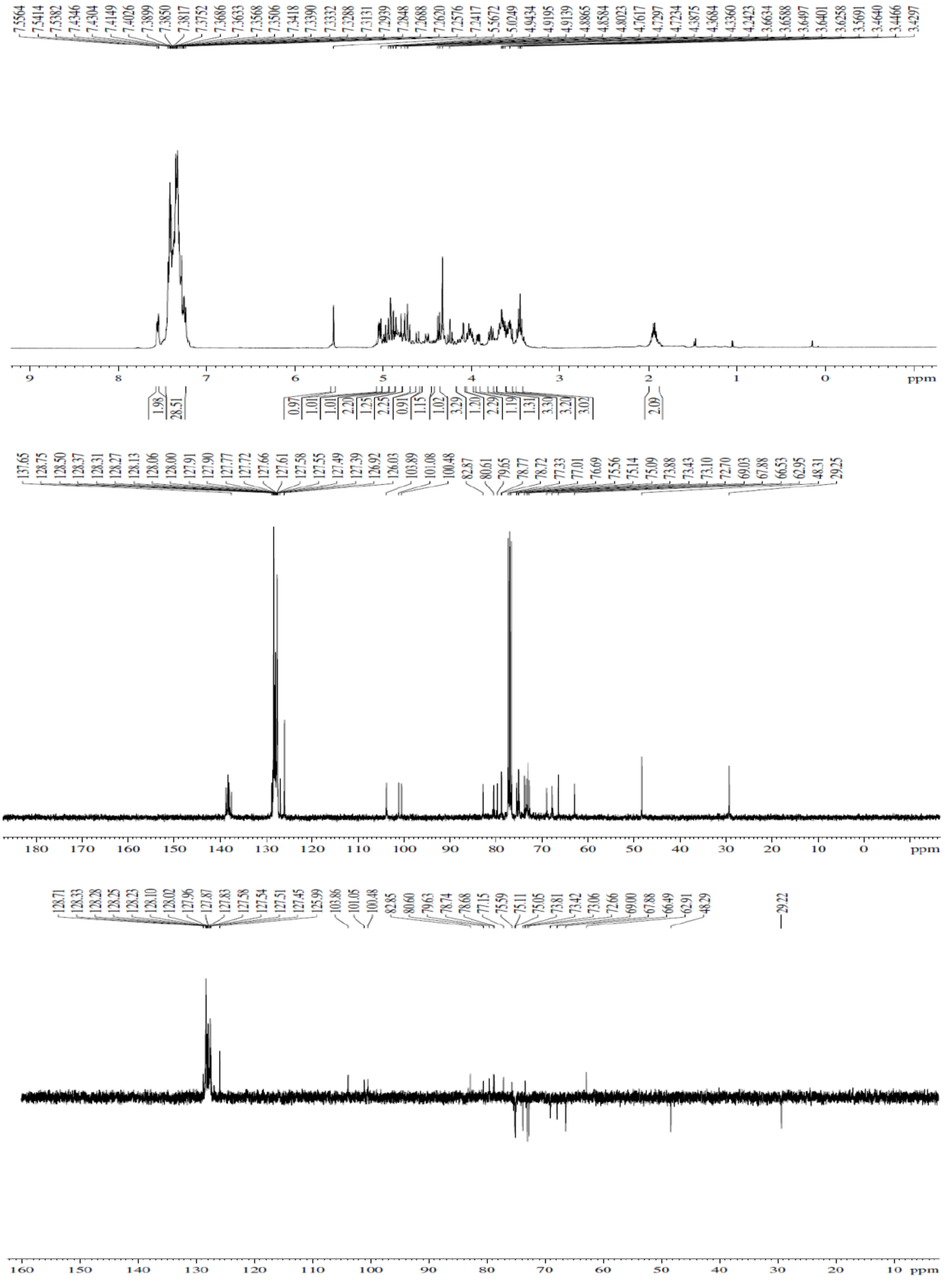

^{1}H, ^{13}C and DEPT 135 NMR spectra of 3-azidopropyl (2,3-di-*O*-benzyl-4,6-*O*-benzylidene-β-D-glucopyranosyl)-(1→4)-2,3,6-tri-*O*-benzyl-β-D-galactopyranoside (**8**) ($CDCl_3$).

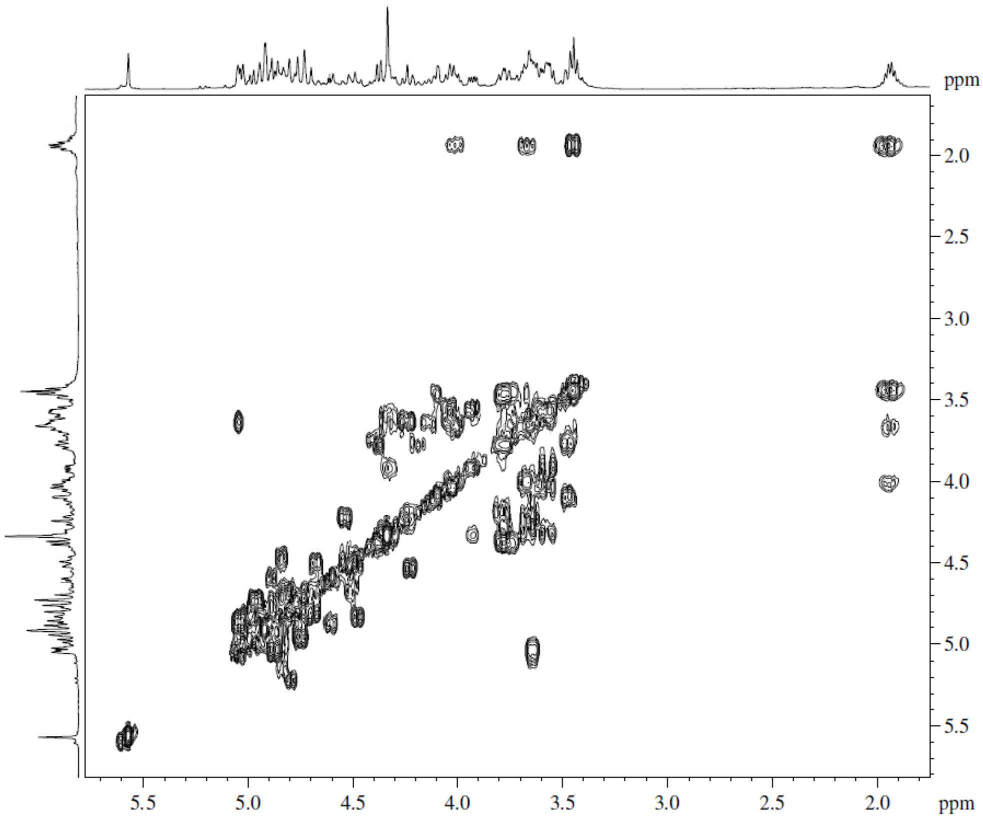

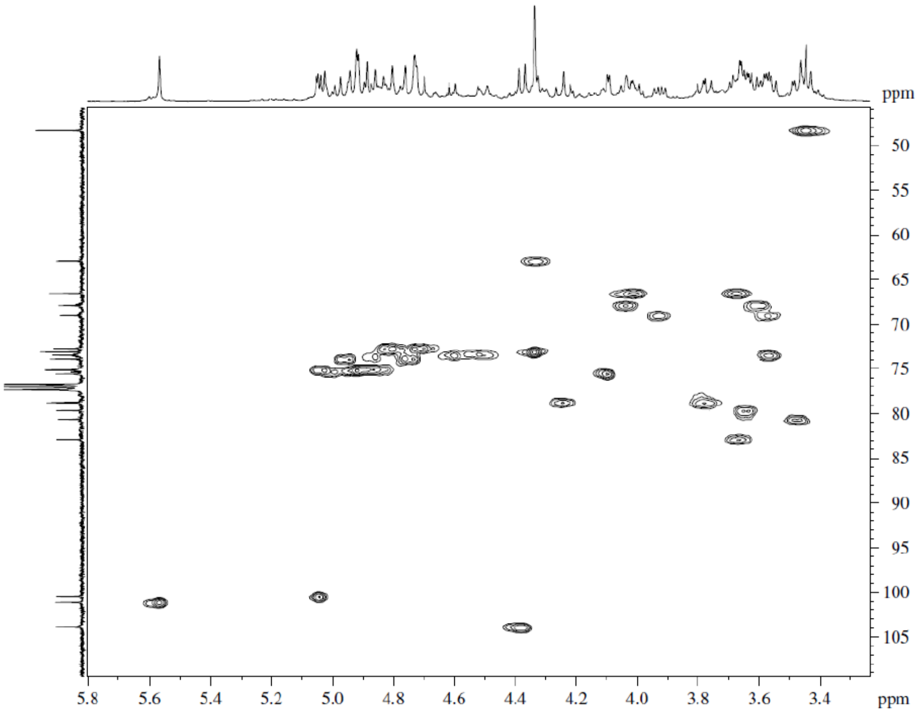

2D COSY and HSQC NMR spectra (selected regions) of 3-azidopropyl (2,3-di-*O*-benzyl-4,6-*O*-benzylidene-β-D-glucopyranosyl)-(1→4)-2,3,6-tri-*O*-benzyl-β-D-galactopyranoside (**8**) ($CDCl_3$).

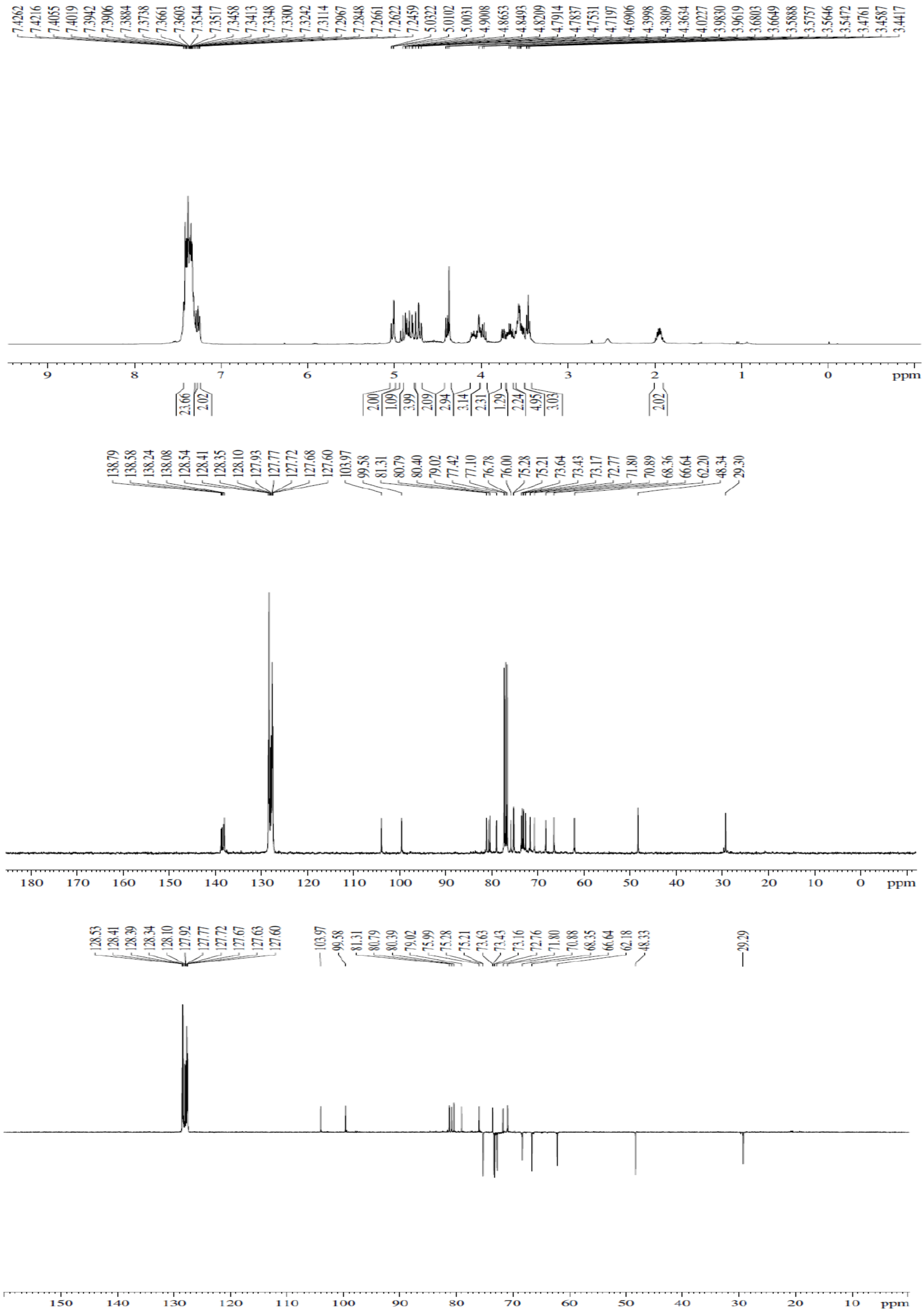

^{1}H, ^{13}C and DEPT 135 NMR spectra of 3-azidopropyl (2,3-di-*O*-benzyl-β-D-glucopyranosyl)-(1→4)-2,3,6-tri-*O*-benzyl-β-D-galactopyranoside (**9**) ($CDCl_3$).

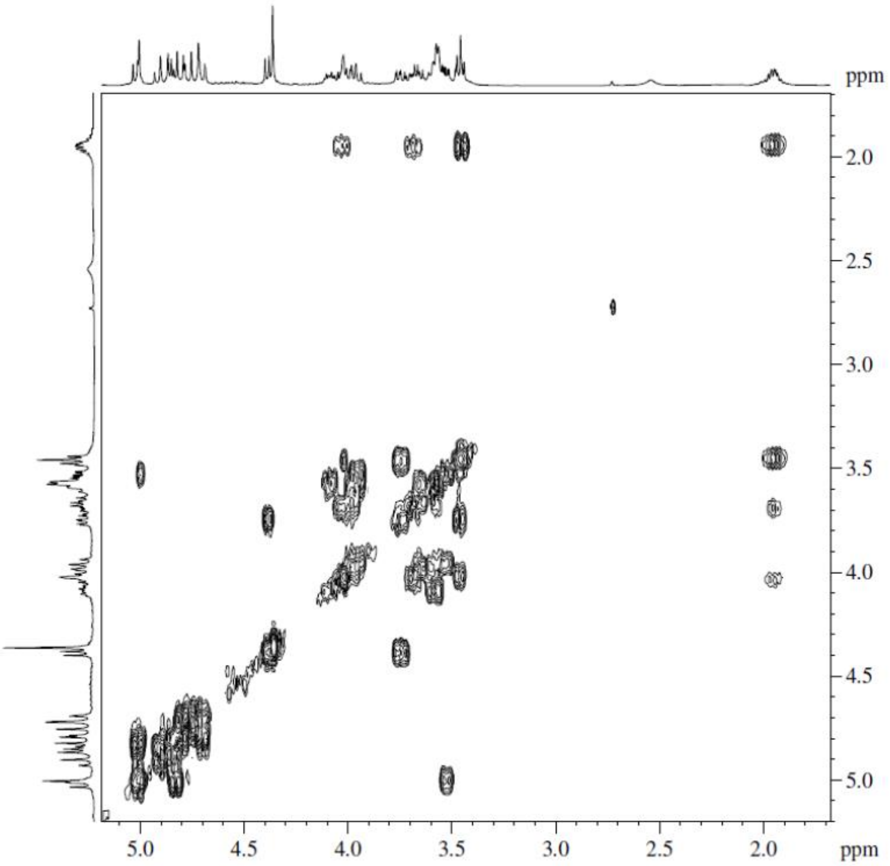

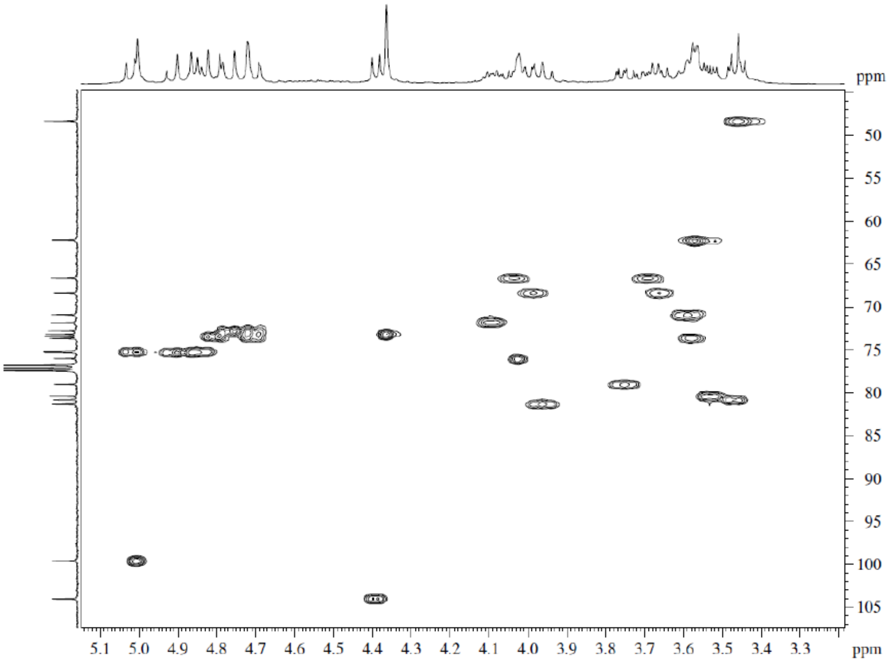

2D COSY and HSQC NMR spectra (selected regions) of 3-azidopropyl (2,3-di-*O*-benzyl-β-D-glucopyranosyl)-(1→4)-2,3,6-tri-*O*-benzyl-β-D-galactopyranoside (**9**) ($CDCl_3$).

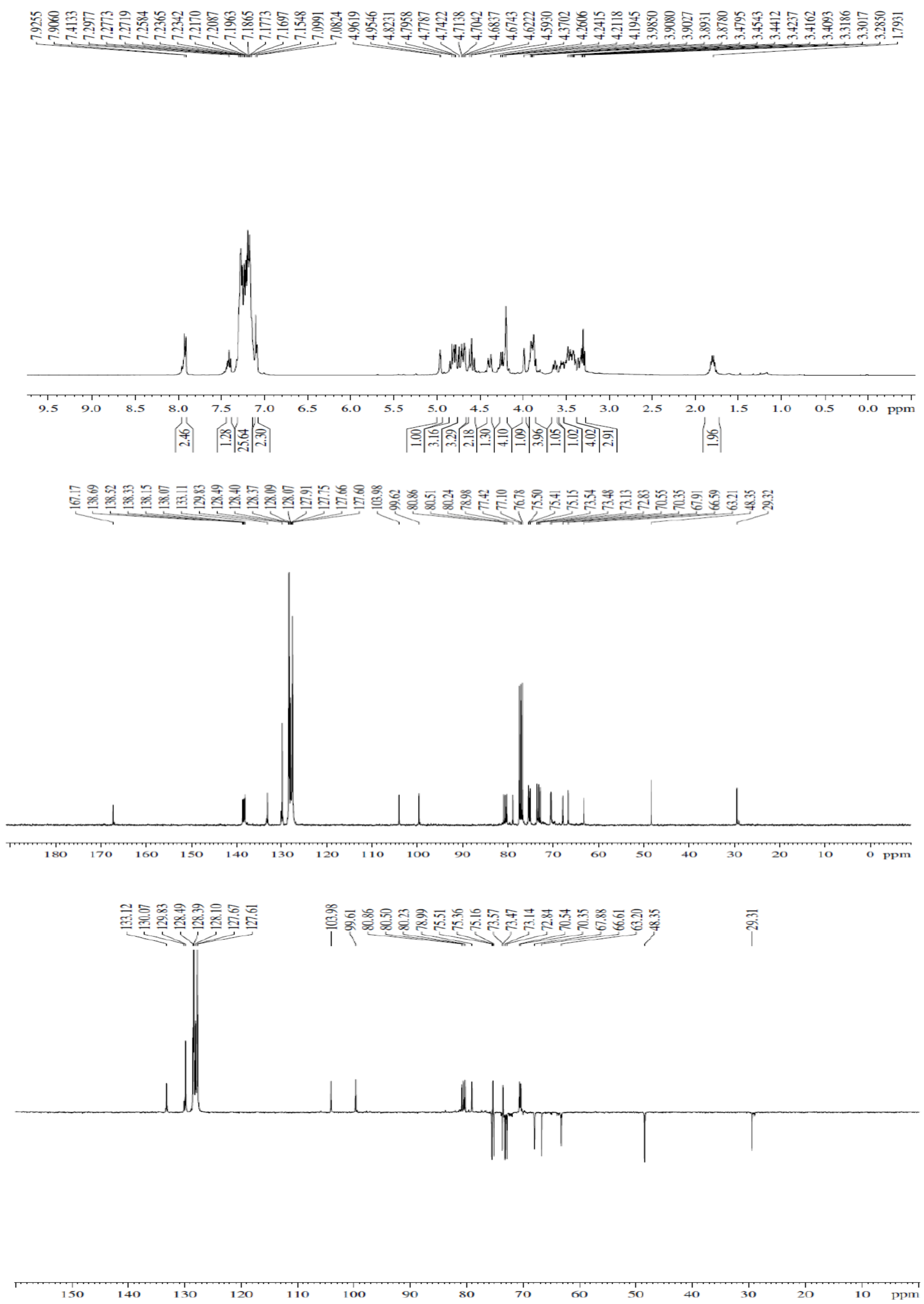

^{1}H, ^{13}C and DEPT 135 NMR spectra of 3-azidopropyl (6-*O*-benzoyl-2,3-di-*O*-benzyl-β-D-glucopyranosyl)-(1→4)-2,3,6-tri-*O*-benzyl-β-D-galactopyranoside (**10**) ($CDCl_3$).

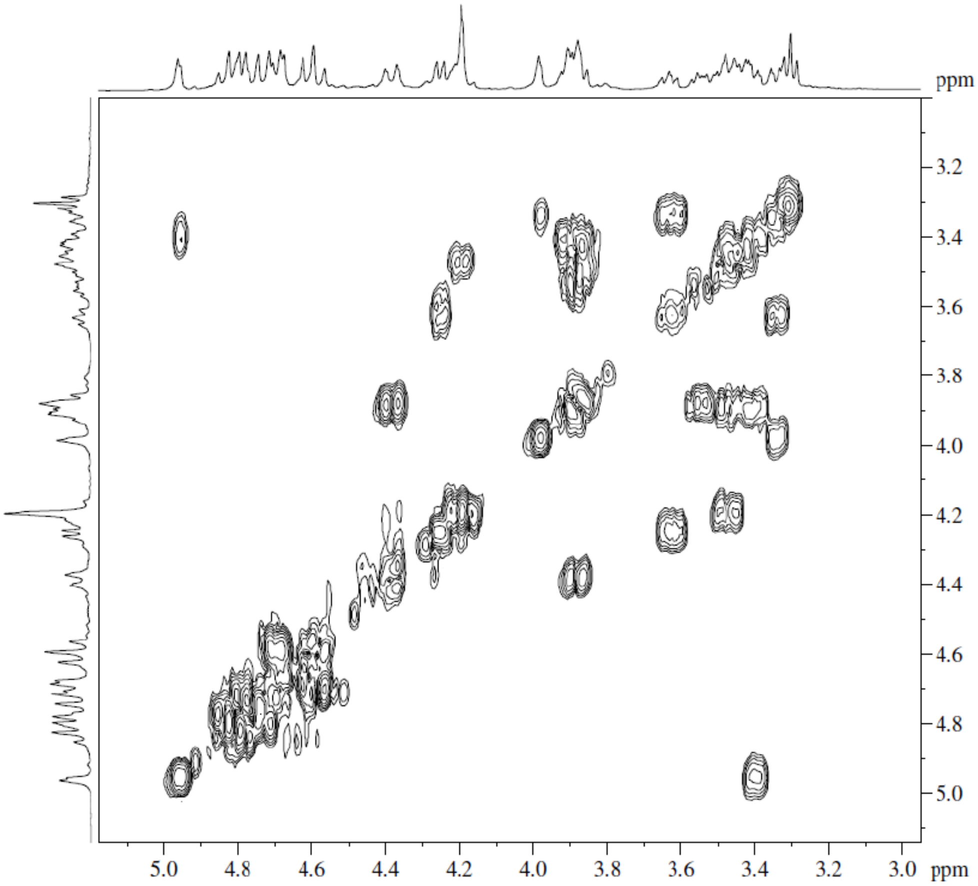

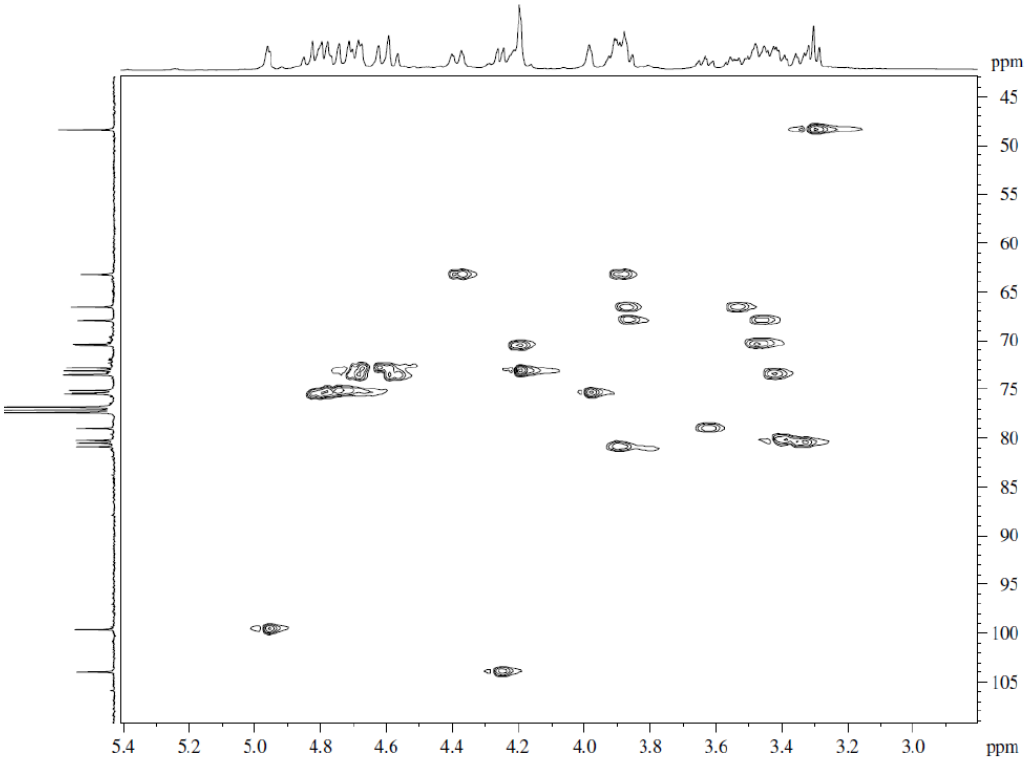

2D COSY and HSQC NMR spectra (selected regions) of 3-azidopropyl (6-*O*-benzoyl-2,3-di-*O*-benzyl-β-D-glucopyranosyl)-(1→4)-2,3,6-tri-*O*-benzyl-β-D-galactopyranoside (**10**) ($CDCl_3$).

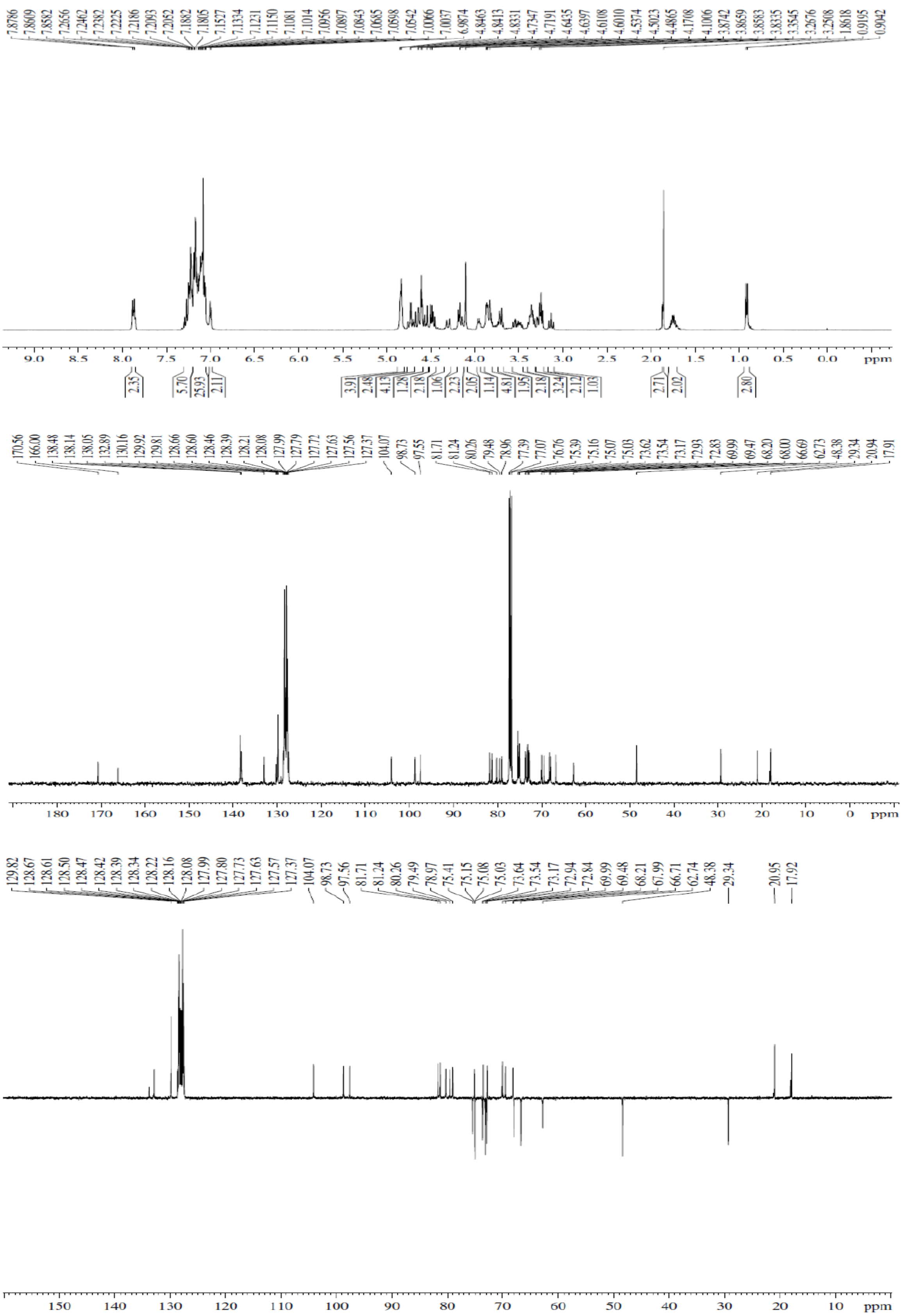

^{1}H, ^{13}C and DEPT 135 NMR spectra of 3-azidopropyl (2-*O*-acetyl-4-*O*-benzyl-α-L-rhamnopyranosyl)-(1→4)-(6-*O*-benzoyl-2,3-di-*O*-benzyl-β-D-glucopyranosyl)-(1→4)-2,3,6-tri-*O*-benzyl-β-D-galactopyranoside (**11**) ($CDCl_3$).

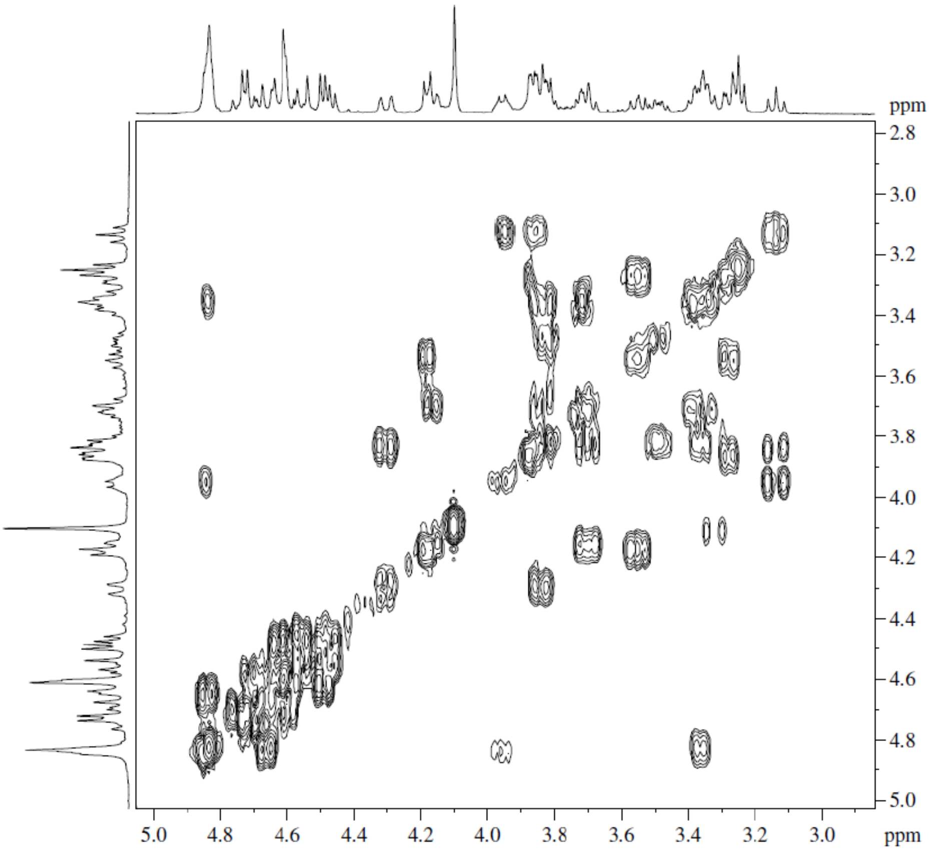

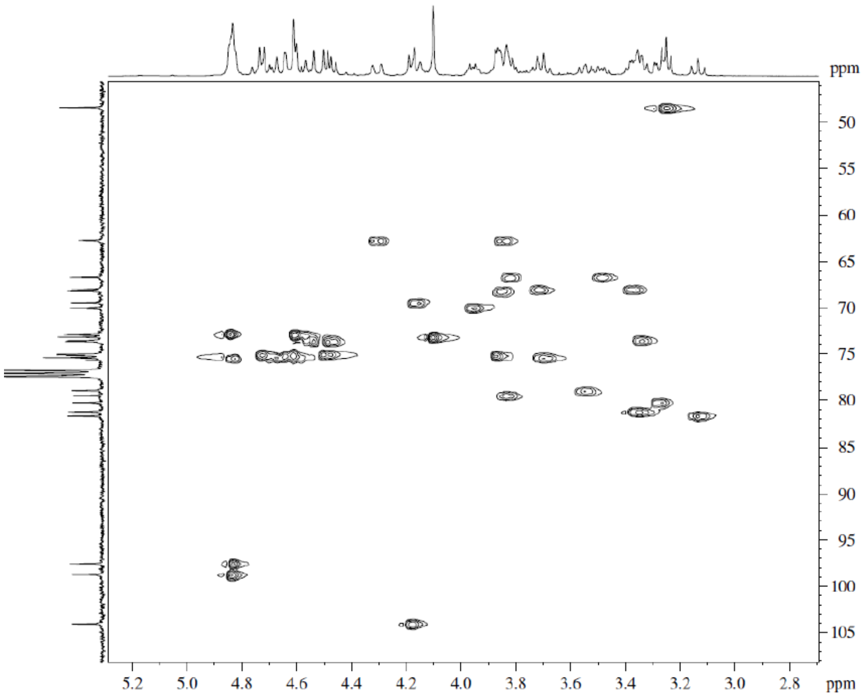

2D COSY and HSQC NMR spectra (selected regions) of 3-azidopropyl (2-*O*-acetyl-4-*O*-benzyl-α-L-rhamnopyranosyl)-(1→4)-(6-*O*-benzoyl-2,3-di-*O*-benzyl-β-D-glucopyranosyl)-(1→4)-2,3,6-tri-*O*-benzyl-β-D-galactopyranoside (**11**) ($CDCl_3$).

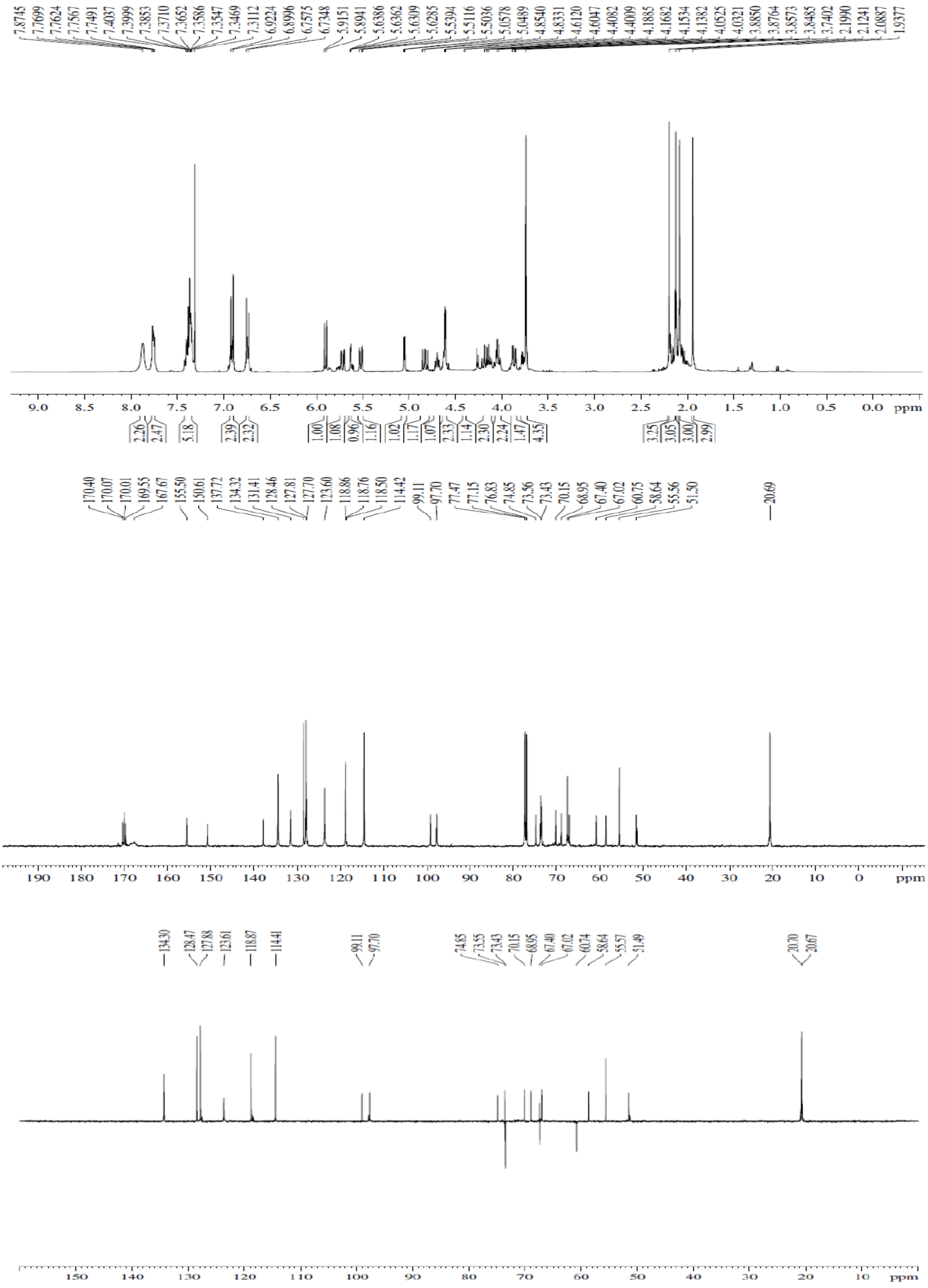

^{1}H, ^{13}C and DEPT 135 NMR spectra of *p*-methoxyphenyl (3,4,6-tri-*O*-acetyl-2-azido-2-deoxy-α-D-galactopyranosyl)-(1→4)-(3-*O*-acetyl-6-*O*-benzyl-2-deoxy-2-phthalimido-β-D-galactopyranoside (**12**) ($CDCl_3$).

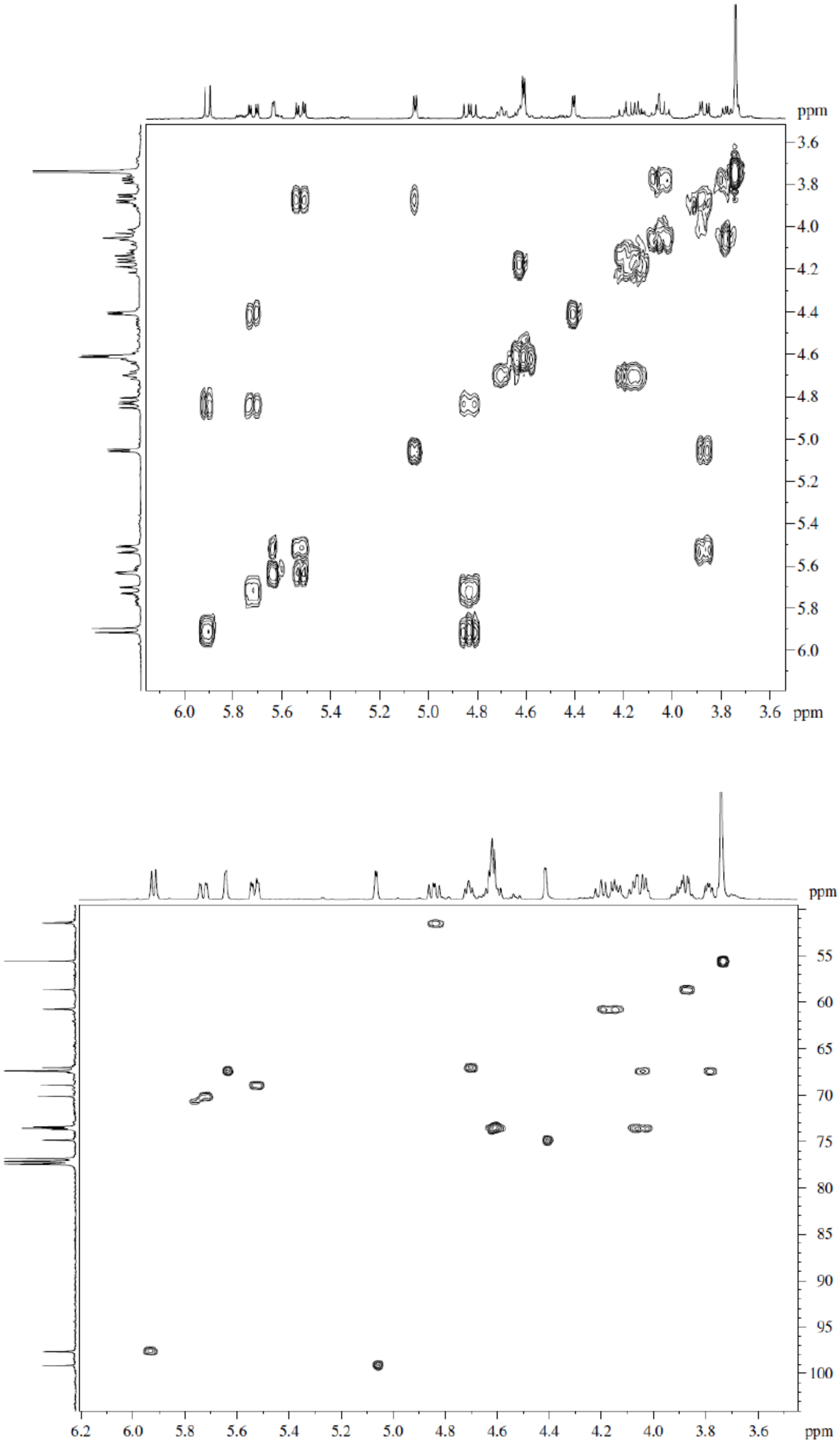

2D COSY and HSQC NMR spectra (selected regions) of *p*-methoxyphenyl (3,4,6-tri-*O*-acetyl-2-azido-2-deoxy-α-D-galactopyranosyl)-(1→4)-(3-*O*-acetyl-6-*O*-benzyl-2-deoxy-2-phthalimido-β-D-galactopyranoside (**12**) ($CDCl_3$).

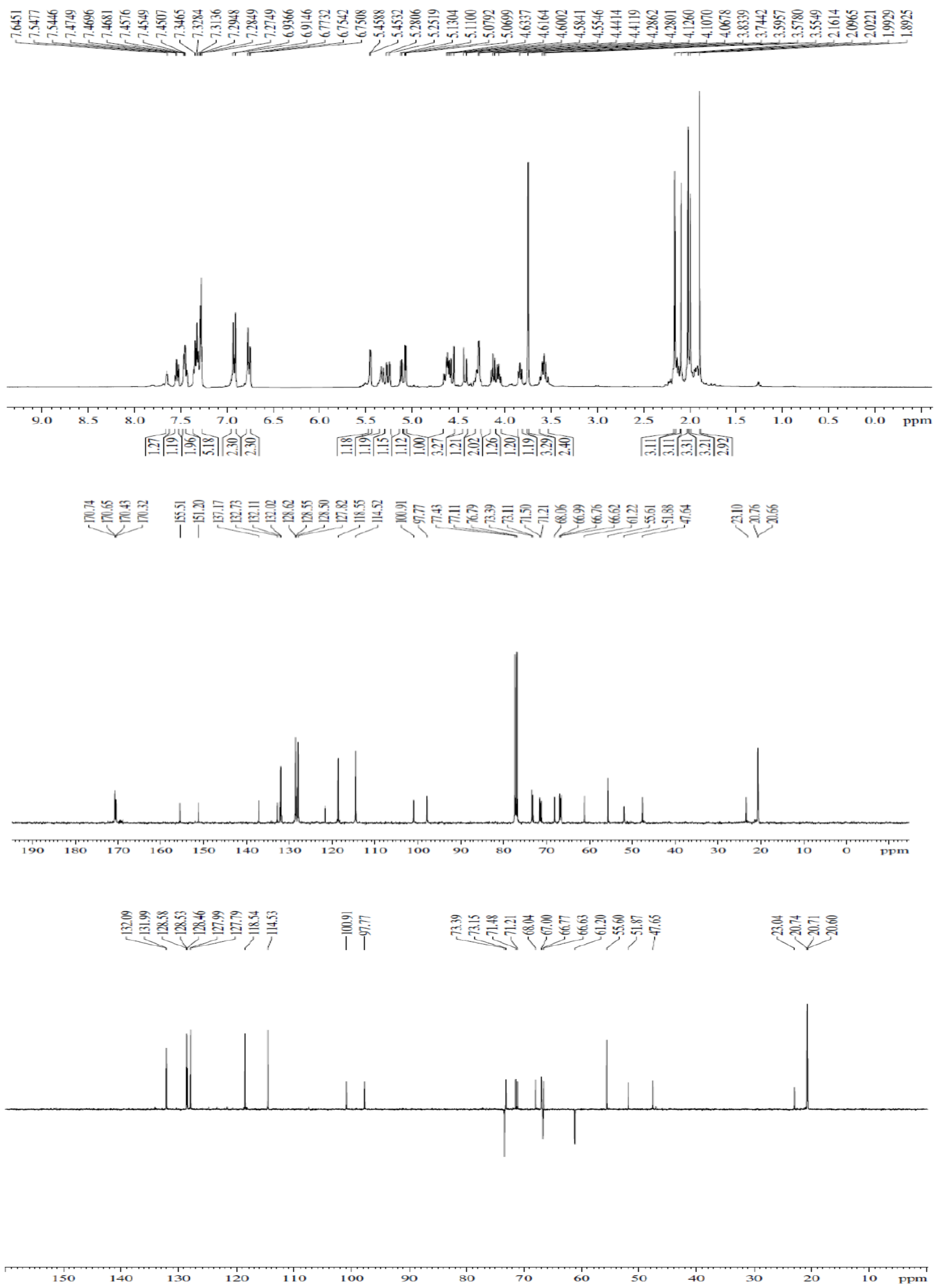

^{1}H, ^{13}C and DEPT 135 NMR spectra of *p*-methoxyphenyl (2-acetamido-3,4,6-tri-*O*-acetyl-2-deoxy-α-D-galactopyranosyl)-(1→4)-(3-*O*-acetyl-6-*O*-benzyl-2-deoxy-2-phthalimido-β-D-galactopyranoside (**13**) ($CDCl_3$).

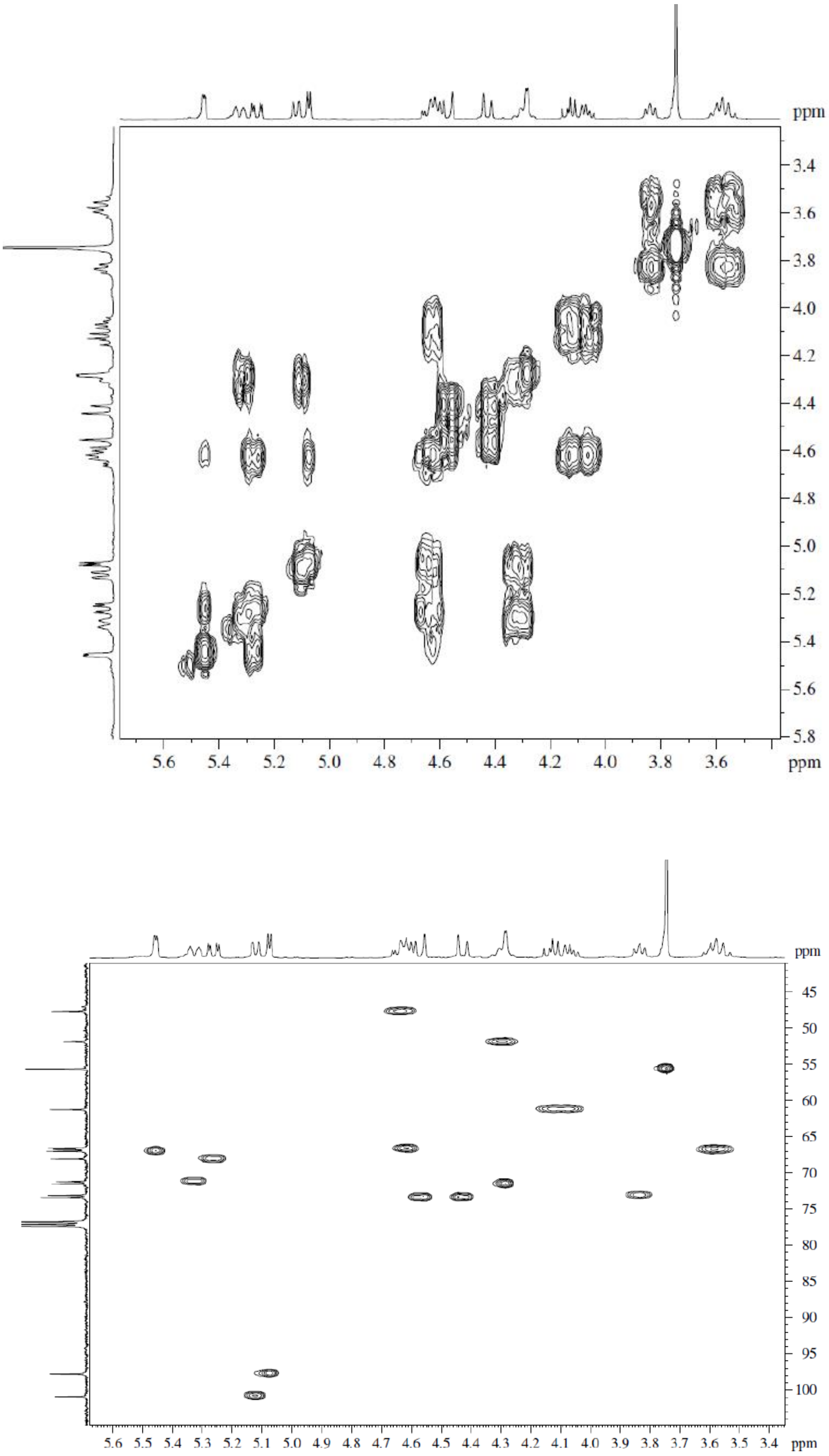

2D COSY and HSQC NMR spectra (selected regions) of *p*-methoxyphenyl (2-acetamido-3,4,6-tri-*O*-acetyl-2-deoxy-α-D-galactopyranosyl)-(1→4)-(3-*O*-acetyl-6-*O*-benzyl-2-deoxy-2-phthalimido-β-D-galactopyranoside (**13**) ($CDCl_3$).

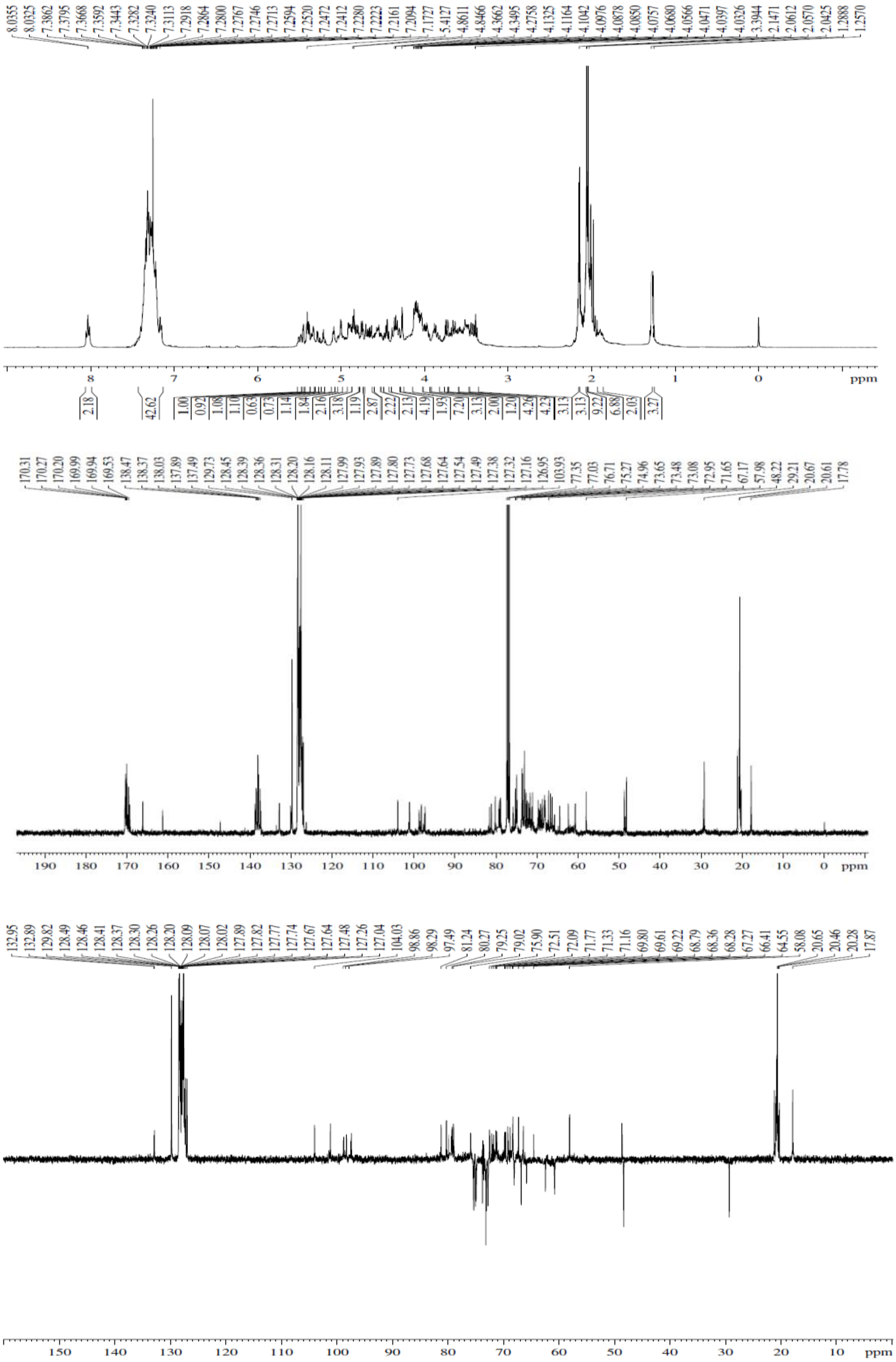

^{1}H, ^{13}C and DEPT 135 NMR spectra of 3-azidopropyl (2-acetamido-3,4,6-tri-*O*-acetyl-2-deoxy-α-D-galactopyranosyl)-(1→4)-(3-*O*-acetyl-6-*O*-benzyl-2-deoxy-2-phthalimido-β-D-galactopyranosyl)-(1→3)-(2-*O*-acetyl-4-*O*-benzyl-α-L-rhamnopyranosyl)-(1→4)-(6-*O*-benzoyl-2,3-di-*O*-benzyl-β-D-glucopyranosyl)-(1→4)-2,3,6-tri-*O*-benzyl-β-D-galactopyranoside (**15**) ($CDCl_3$).

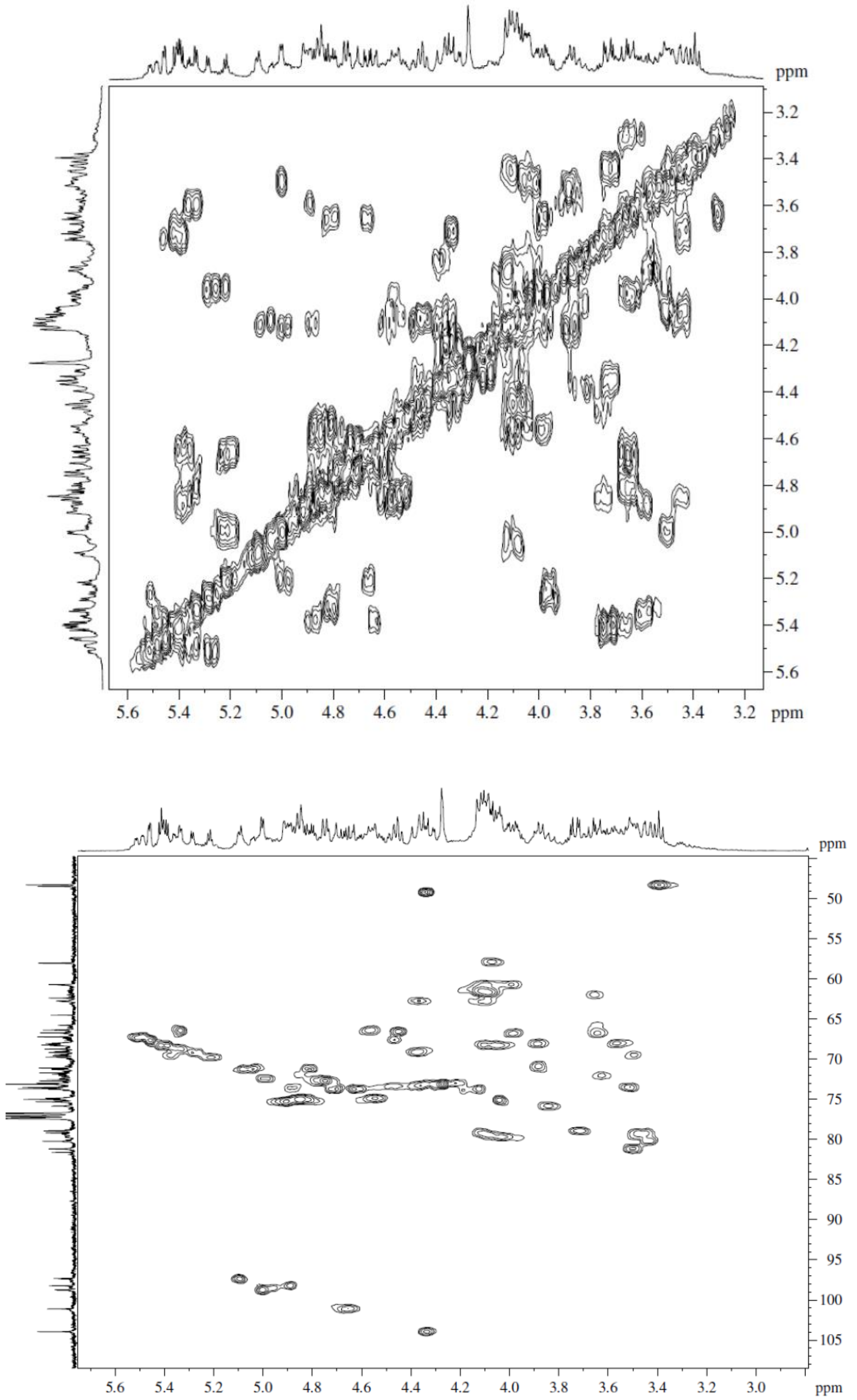

2D COSY and HSQC NMR spectra (selected regions) of 3-azidopropyl (2-acetamido-3,4,6-tri-*O*-acetyl-2-deoxy-α-D-galactopyranosyl)-(1→4)-(3-*O*-acetyl-6-*O*-benzyl-2-deoxy-2-phthalimido-β-D-galactopyranosyl)-(1→3)-(2-*O*-acetyl-4-*O*-benzyl-α-L-rhamnopyranosyl)-(1→4)-(6-*O*-benzoyl-2,3-di-*O*-benzyl-β-D-glucopyranosyl)-(1→4)-2,3,6-tri-*O*-benzyl-β-D-galactopyranoside (**15**) ($CDCl_3$).
